# The common YAP activation mediates corneal epithelial regeneration and repair with different-sized wounds

**DOI:** 10.1038/s41536-021-00126-2

**Published:** 2021-03-26

**Authors:** Yijian Li, Lingling Ge, Xia Chen, Yumei Mao, Xianliang Gu, Bangqi Ren, Yuxiao Zeng, Min Chen, Siyu Chen, Jinhua Liu, Yuli Yang, Haiwei Xu

**Affiliations:** 1 Southwest Hospital/Southwest Eye Hospital, Third Military Medical University (Army Medical University), Chongqing, China; 2 Key Lab of Visual Damage and Regeneration & Restoration of Chongqing, Chongqing, China; 3 Southwest University, Chongqing, China; 4 North Sichuan Medical College, Sichuan, China

**Keywords:** Actin, Regeneration

## Abstract

Regeneration/repair after injury can be endowed by adult stem cells (ASCs) or lineage restricted and even terminally differentiated cells. In corneal epithelium, regeneration after a large wound depends on ASCs (limbal epithelial stem cells, LESCs), whereas repair after a small wound is LESCs-independent. Here, using rat corneal epithelial wounds with different sizes, we show that YAP activation promotes the activation and expansion of LESCs after a large wound, as well as the reprogramming of local epithelial cells (repairing epithelial cells) after a small wound, which contributes to LESCs-dependent and -independent wound healing, respectively. Mechanically, we highlight that the reciprocal regulation of YAP activity and the assembly of cell junction and cortical F-actin cytoskeleton accelerates corneal epithelial healing with different-sized wounds. Together, the common YAP activation and the underlying regulatory mechanism are harnessed by LESCs and lineage-restricted epithelial cells to cope with corneal epithelial wounds with different sizes.

## Introduction

Adult stem cells (ASCs) have the unique capacity to both self-renew and differentiate into all cell types of their tissues. It is commonly believed that resident ASCs are essential for tissue homeostasis and regeneration/repair after injury. However, regeneration/repair can also be endowed by lineage restricted and even terminally differentiated cells, such as hepatocytes in liver^1,2^. Furthermore, when loss of resident ASCs, lineage-restricted progenitors and even terminally differentiated cells can dedifferentiate or reprogram to restore ASCs pools, such as secretory and enterocyte precursors in intestinal crypt^3,4^ and club cells in lung airway epithelium^5^. Obviously, organs and tissues that exhibit divergent biological properties and are subject to different biological and physical challenges harness diverse mechanisms for endogenous regeneration and repair^6,7^.

The function of the cornea is largely dependent on the maintenance of a healthy stratified epithelium, which is replenished by corneal epithelial ASCs (limbal epithelial stem cells, LESCs) located in limbus, the transition zone between the cornea and conjunctiva^8^. According to LESCs hypothesis, LESCs give rise to transit-amplifying cells (TACs), which rapidly proliferate, centripetally migrate and gradually leave the basal layer (basal cells) vertically through the suprabasal layers (wing cells) to the superficial layers (squamous cells), where they terminally differentiate and are shed from the ocular surface^9^. LESCs slowly divide during normal homeostasis, and become more active and rapidly proliferate to accelerate regeneration after a large wound (hereafter, defined as LESCs-dependent regeneration). However, a small wound of the central cornea is repaired independent of LESCs activation (hereafter, defined as LESCs-independent repair)^10–12^. It is still unclear that what factors determine these different strategies or whether a common mechanism is shared by LESCs-dependent regeneration and LESCs-independent repair after wounds with different sizes.

The Hippo pathway is a highly conserved signaling pathway implicated in development, homeostasis, regeneration, and diseases^13^. The canonical Hippo kinase cascade is initiated by MST1/2, which phosphorylates and activates LATS1/2. The activated LATS1/2 then inactivates YAP and TAZ by repressing their nuclear translocation via phosphorylation (e.g., YAP Ser127). When upstream signals are low, YAP/TAZ enters into the nucleus, interacts with transcription factors (e.g., TEADs), and drives or represses the expression of target genes^14^. The link to a diverse array of upstream inputs (including cell polarity and junctions, cytoskeleton and mechanical cues, metabolic cues and stress signals) and wide cross-talks with other signaling pathways (e.g., Wnt, Notch, TGF/BMP, and inflammatory signal) make Hippo pathway to be a key sensor for tissue integrity and can directly respond to injury, where YAP/TAZ integrates multiple signals to regulate cell proliferation, plasticity, and fate determination that are essential for tissue regeneration/repair^15,16^.

Here, we investigate LESCs-dependent regeneration of corneal epithelium after a large wound (4 mm diameter) and LESCs-independent repair after a small wound (1.5 mm diameter) on adult rat central cornea. We show that YAP activation mediates the activation and expansion of LESCs to seal the defected epithelium after a large wound, whereas transient activation of YAP locally reprograms repairing epithelial cells and mediates the LESCs-independent repair after a small wound. Mechanically, using RNA sequencing and genome-wide cDNA microarray analysis, pharmacological treatments, and overexpression/knockdown of YAP with cell co-cultures, we show that disrupted cortical F-actin cytoskeleton and enhanced actin dynamics by inhibition of ROCK/LIMK/Cofilin pathway relieve the suppression on YAP activity. In turn, YAP regulates the assembly of cell junction and cortical F-actin cytoskeleton of epithelial cells, indicating the formation of a regulatory loop. Together, the common YAP activation and the underlying regulatory mechanism are harnessed by LESCs and lineage-restricted epithelial cells to cope with corneal epithelial wounds with different sizes, which provides new insights into the understanding of the nature of regeneration and repair.

## Results

### Activation of YAP during corneal epithelial regeneration after large wound

**Fig. 1:**
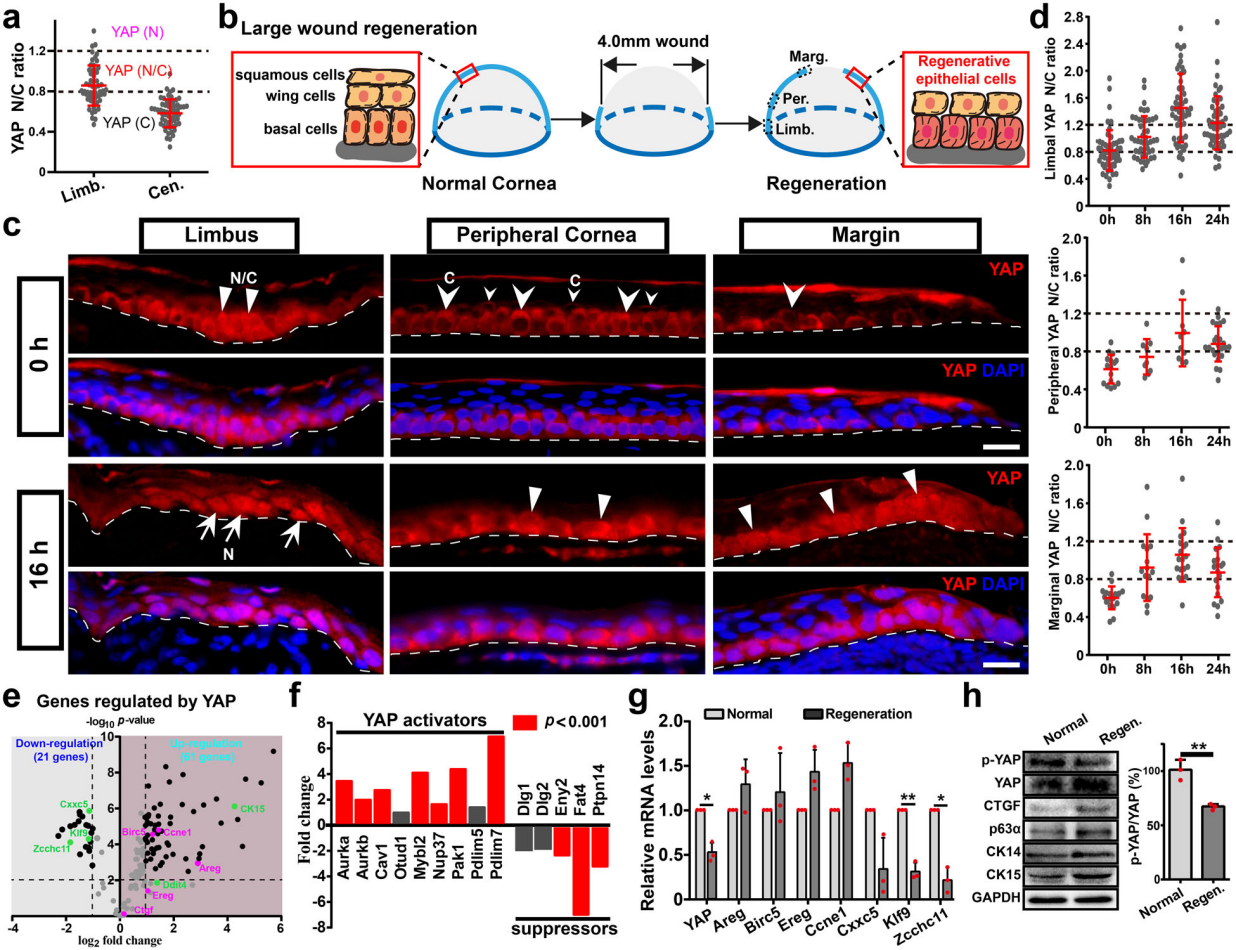
Activation of YAP during corneal epithelial regeneration after large wound. **a** The YAP nuclear/cytoplasmic (N/C) ratio of epithelial cells in limbus and central cornea of adult rat. The YAP subcellular distribution is classified and defined as cytoplasmic (C), nuclear (N), and evenly nuclear and cytoplasmic (N/C). **b** In vivo corneal epithelial regeneration after a large wound (4 mm diameter). Epithelial cells in limbus (Limb.), peripheral cornea (Per.) and regenerative margin (Marg.) are examined. The peripheral and regenerative marginal epithelial cells are defined as regenerative epithelial cells. **c** Immunofluorescence localization of YAP in limbus, peripheral cornea, and regenerative margin at 0 and 16 h post wound (pw). Arrowheads point cytoplasmic YAP; arrows point nuclear YAP; triangles point N/C YAP. The broken white lines identify the epithelial/stromal boundary. Scale bars, 20 μm. **d** The YAP N/C ratio of epithelial cells in limbus, peripheral cornea, and regenerative margin at 0, 8, 16, and 24 h pw. **e** The YAP target genes among the differentially expressed genes during regeneration from published genome-wide cDNA microarray data. Pink dots indicate target genes that driven by YAP; green dots indicate target genes repressed by YAP. **f** The fold changes of mRNA levels of YAP regulators (activators and suppressors) during regeneration relative to normal corneal epithelium. **g** RT-qPCR analysis of YAP and its target genes in normal corneal epithelium and regenerative epithelial cells at 16 h after large wound. **h** Western analysis of indicated proteins in normal corneal epithelium and regenerative epithelial cells (Regen.) at 16 h after large wound. The p-YAP/YAP is quantified and normalized. Data are the mean ± SD, *n* = 3 biological replicates each (**g**, **h**); *p < 0.05, **p < 0.01 (Student’s two-tailed paired *t*-test).

Previous studies indicated that YAP was required for the maintenance of corneal epithelial progenitor cells, and YAP knockdown was not compensated by TAZ^17,18^. Thus, the role of YAP, but not TAZ, is investigated in corneal epithelial regeneration/repair after injury in this study. We first examine the expression and intracellular distribution of YAP, which is classified as predominantly nuclear (N), predominantly cytoplasmic (C), or evenly nuclear and cytoplasmic (N/C). In addition to junctional YAP staining, N/C YAP is also detected in limbal epithelial cells; junctional and cytoplasmic YAP is observed in corneal epithelial basal cells and some wing cells. Most wing cells and all superficial squamous cells do not express YAP (Supplementary Fig. 1a). The lower YAP nuclear/cytoplasmic (N/C) ratio in corneal epithelial cells indicates suppressed YAP transcriptional activity in these epithelial cells (Fig. 1a). Large wound (4 mm diameter) on adult rat cornea is performed to examine the role of YAP in LESCs-dependent regeneration (Fig. 1b). Translocation of YAP into the nucleus is observed in limbal and regenerating epithelial cells at 16 and 24 h post wound (pw) (Fig. 1c; Supplementary Fig. 1b). The YAP N/C ratio increases from 8 to 16 h pw, then decreases at 24 h pw in limbal and regenerating epithelial cells (Fig. 1d). Limbal epithelial cells always show the highest YAP N/C ratio, and YAP N/C ratios of the margin are higher than peripheral epithelial cells from 8 to 24 h pw, especially at 8 h pw (Supplementary Fig. 1c). In addition, p-YAP (Ser127, inactive YAP) staining shows lower fluorescence intensity in limbal epithelial cells (Supplementary Fig. 1d), and the protein level of p-YAP (Ser127) and the p-YAP/YAP ratio decreases during regeneration (Fig. 1h). These results indicate that YAP is re-activated during corneal epithelial regeneration after a large wound.

To further confirm the activation of YAP during regeneration after a large wound, we re-analyze previously published genome-wide cDNA microarray data^19^. 142 YAP target genes are expressed in corneal epithelium. Among these genes, 82 genes are differentially expressed during corneal epithelial regeneration, with 21 genes downregulated and 61 genes upregulated (Fig. 1e; Supplementary Table 1). A large number of canonical YAP transcriptional target genes, e.g., Birc5, Areg, Ereg, Ccne1, and CTGF, are among the upregulated genes (Fig. 1e, g; Supplementary Fig. 1e). As YAP also functions as transcriptional co-repressor^20,21^, target genes repressed by YAP, e.g., Cxxc5, Klf9, and Zcchc11, are downregulated during regeneration (Fig. 1e, g). Interestingly, Ddit4 and CK15 (known as a LESCs marker), which are repressed by YAP in the previous analysis^20^, are upregulated during corneal epithelial regeneration (Fig. 1e, h; Supplementary Fig. 3d). This is in agreement with the notion that YAP/TAZ acts as co-activator or co-repressor of gene expression, depending on cell type and conditions^20,21^. Furthermore, activators of YAP (e.g., Aurka/b, Cav1, Otud1, Pdlim5/7) are upregulated, whereas suppressors of YAP (e.g., Ptpn14, Eny2) are downregulated during regeneration (Fig. 1f; Supplementary Table 2). Interestingly, the mRNA level of YAP decreased during regeneration (Fig. 1g). Together with the decreased protein level of p-YAP (Ser127) and the p-YAP/YAP ratio, these results indicate post-transcriptional and even post-translational mechanisms of the YAP activation. Thus, our analysis of YAP nuclear localization, YAP phosphorylation state, and levels of YAP and its target genes and activators/suppressors conclusively suggest that YAP is re-activated in limbal and regenerating epithelial cells during corneal epithelial regeneration.

### YAP mediates LESCs-dependent regeneration through regulating proliferation and migration

**Fig. 2:**
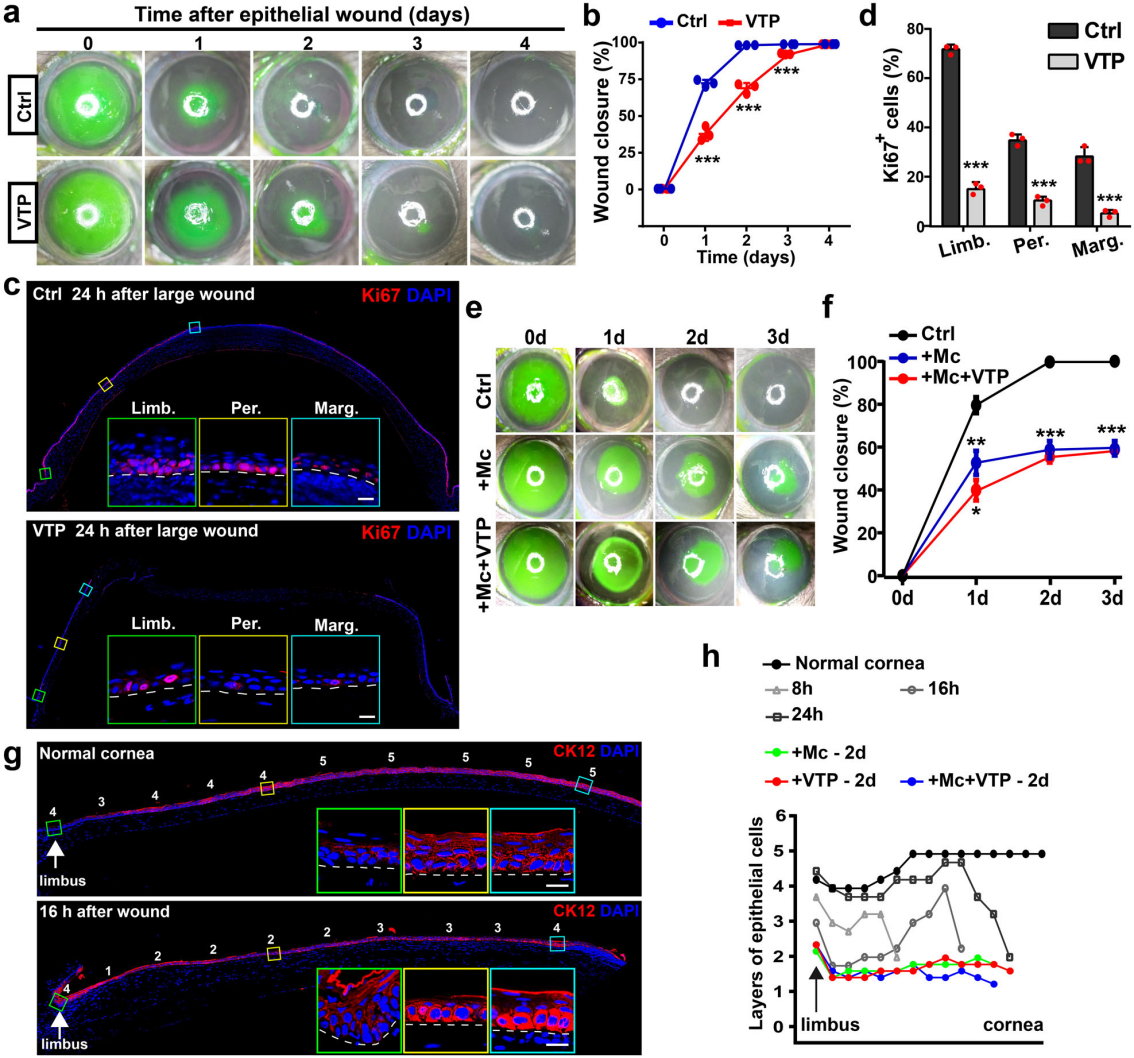
YAP activation mediates LESCs-dependent regeneration through proliferation and migration. **a**, **b** After the scrape of 4mm-diameter corneal epithelium, corneas are treated with vehicle (DMSO, Ctrl) or VTP. The corneal epithelial defects are stained with fluorescein sodium at 0, 1, 2, 3, and 4 days after wound. Quantifications of wound closure (%) at 0, 1, 2, 3, and 4 days. **c**, **d** Proliferation (Ki67-positive) of regenerative epithelial cells at 24 h pw without or with VTP treatment. Quantification of percentages of Ki67-positive regenerative epithelial cells at 24 h pw without or with VTP treatment. **e**, **f** The corneal epithelial defects are stained with fluorescein sodium at 0, 1, 2, and 3 days after 4 mm-diameter wound under indicated conditions. Quantifications of wound closure (%) at 0, 1, 2, and 3 days. +Mc vs Ctrl; +Mc+VTP vs +Mc. **g**, **h** CK12 staining of corneal epithelium in normal cornea and during regeneration at 16 h pw. The numbers shown are layers of epithelial cells. Quantifications of epithelial cells layers at indicated time points or under indicated treatments. Scale bars, 20 μm (**c**, **g**). Data are the mean ± SD, *n* = 3 corneas (**b**, **f**), *n* = 3 sections (**d**); Data are the mean from four sections (**h**); *p < 0.05, **p < 0.01, ***p < 0.001 (Student’s two-tailed unpaired *t*-test). Ctrl, 0.1% DMSO; VTP, ver*t*eporfin, 10 μM; Mc, mitomycin C, 5 μg/mL.

We next investigate the contribution of activated YAP to corneal epithelial regeneration after a large wound. In control corneas, corneal epithelial defects are closed in 2 days. However, epithelial defects remain about 30% of total defect sizes at 2 days and are closed at 4 days after treated with verteporfin (VTP), a specific small molecule inhibitor that blocks YAP–TEAD interaction and suppresses their transcriptional activity^22^ (Fig. 2a, b). Previous studies demonstrated that corneal epithelial regeneration was accelerated by limbal population pressure through LESCs proliferation and centripetal migration^23^. Proliferation (Ki67 as a marker) of LESCs and regenerating epithelial cells significantly increases from 8 to 24 h pw compared with at 0 h pw, and limbal epithelial cells always show the highest proliferative rate (Fig. 2c; Supplementary Fig. 2a–d). VTP significantly inhibits the proliferation of LESCs and regenerating epithelial cells (Fig. 2c, d; Supplementary Fig. 2d). Additionally, LESCs markers CK14 and p63α are restricted to the limbus at 0 h pw, whereas CK14 and p63α expand in the limbal epithelial cells (Supplementary Fig. 3a, b), as well as highly expressed in basal regenerative epithelial cells from 8 to 24 h pw during regeneration (Supplementary Fig. 3a–c). RT-qPCR reveals increased mRNA levels of LESCs markers, Abcg2, CK14, and CK15 (Supplementary Fig. 3d), as well as Western blotting reveals increased protein levels of LESCs markers, p63α, CK14, and CK15, during regeneration (Fig. 1h). However, VTP inhibits the upregulated expression of p63α in limbal and regenerative epithelial cells during regeneration (Supplementary Fig. 3e, f).

Surprisingly, inhibition of cell proliferation by mitomycin C (Mc), a DNA cross-linking agent, does not stop the closure of epithelial defects. About 50% of total defect sizes are closed at 1 day pw after Mc treatment. However, the epithelial defects won’t be sealed even at 3 days pw, and the percentage of wound closure remains at about 58% (Fig. 2e, f). This indicates that centripetal cell migration of residual epithelium also contributes to the wound closure, at least at the early stage. As expected, the superficial and even suprabasal layers (squamous and wing cells) of peripheral epithelium are lost during regeneration (Fig. 2g, h), accompanying with few TUNEL+ apoptotic epithelial cells (Supplementary Fig. 2e). These data indicate that centripetal migration, but not elevated apoptosis, results in the loss of superficial and suprabasal epithelium. Furthermore, VTP further delays wound closure and decelerates the centripetal migration of peripheral epithelium after Mc treatment (Fig. 2e, f, h). Together, these data suggest that YAP activation contributes to corneal epithelial regeneration through regulating the proliferation and expansion of LESCs, as well as centripetal migration of superficial and suprabasal epithelial cells of peripheral epithelium.

### YAP-mediated reprogramming of repairing epithelial cells contributes to LESCs-independent repair after small wound

**Fig. 3:**
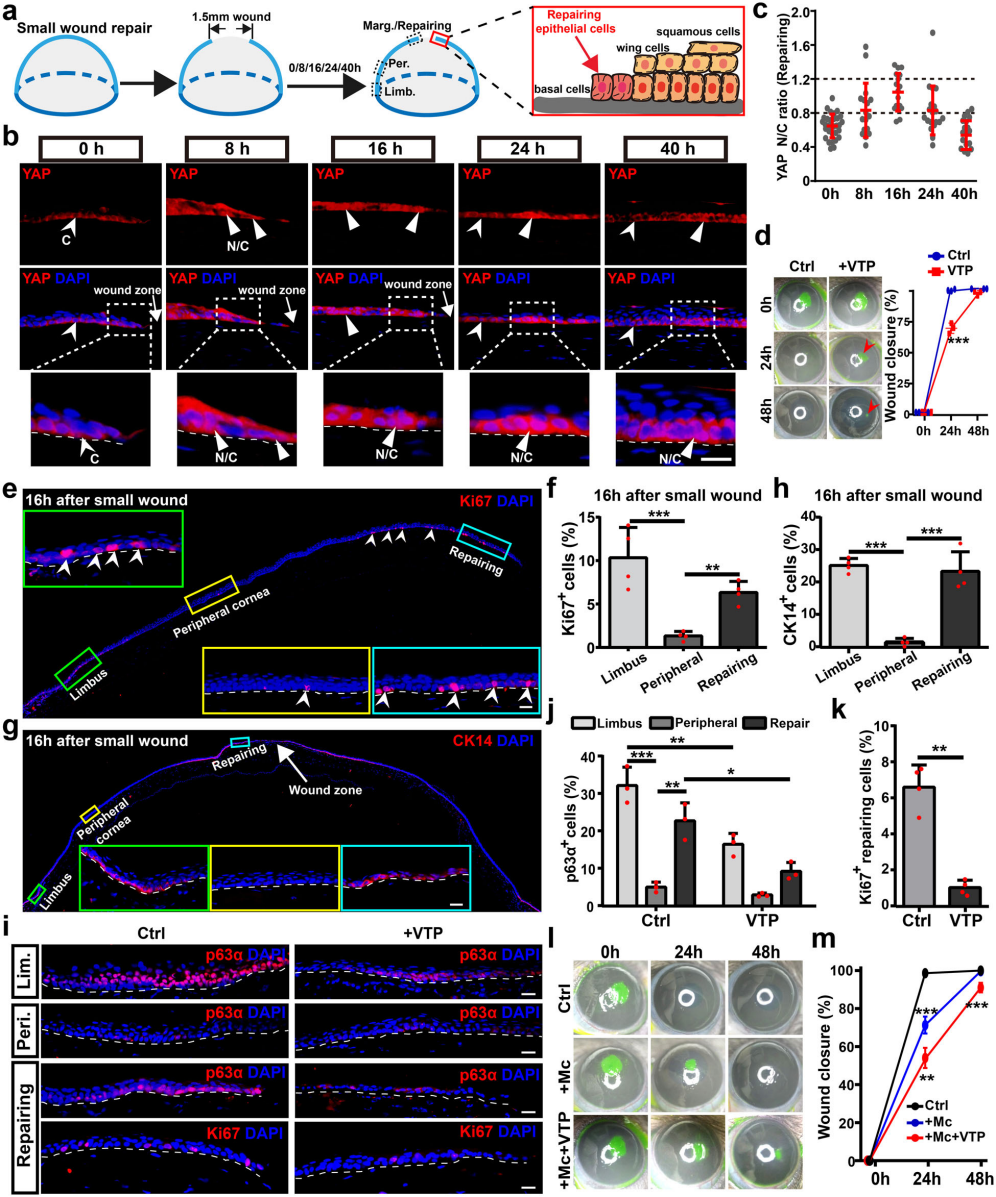
YAP-mediated reprogramming of repairing epithelial cells contributes to LESCs-independent repair after small wound. **a** In vivo corneal epithelial repair after a small wound (1.5 mm diameter). To distinguish from regenerative epithelial cells during regeneration after a large wound, these activated epithelial cells near the small wound are defined as repairing epithelial cells. **b** Immunofluorescence localization of YAP in repairing epithelial cells at 0, 8, 16, 24, and 40 h pw. **c** The YAP N/C ratio of repairing epithelial cells at 0, 8, 16, 24, and 40 h pw. **d** The corneal epithelial defects are stained with fluorescein sodium at 0, 24, and 48 h after small wound under indicated conditions. The arrow points to the remaining epithelial defect. Quantifications of wound closure (%) at 0, 24, and 48 h after small wound. **e**, **f** Proliferation (Ki67-positive) of epithelial cells in limbus, peripheral cornea, and repairing margin during repair at 16 h pw. Quantification of percentages of Ki67-positive proliferative epithelial cells. **g**, **h** Immunofluorescence staining and quantification of LESCs marker CK14 in limbus, peripheral cornea, and repairing margin during repair at 16 h pw. **i**–**k** Immunofluorescence staining and quantification of LESCs marker p63α and proliferative marker Ki67 during repair at 16 h pw in vehicle (Ctrl) and VTP-treated corneas. **l**, **m** The corneal epithelial defects are stained with fluorescein sodium at 0, 24, and 48 h after a small wound under indicated conditions. Quantifications of wound closure (%) at 0, 24, and 48 h after a small wound. +Mc vs Ctrl; +Mc+VTP vs +Mc. Scale bars, 20 μm (**b**, **e**, **g**, **i**). Data are the mean ± SD, *n* = 3 corneas (**d**, **m**); *n* = 4 sections (**f**, **h**, **k**), *n* = 3 sections (**j**); *p < 0.05, **p < 0.01, ***p < 0.001; One-way ANOVA with Dunnett’s post test (**f**, **h**, **j**) or Student’s two-tailed unpaired *t*-test (**d**, **k**, **m**). Ctrl, 0.1% DMSO; VTP, verteporfin, 10 μM; Mc, mitomycin C, 5 μg/mL.

The rapid activation of YAP at the margin at 8 h pw during regeneration implies that wound could activate YAP of surrounding corneal epithelial cells (Supplementary Fig. 1c). Thus, we next explore whether YAP mediates LESCs-independent repair after a small wound (1.5 mm diameter) on adult rat central cornea (Fig. 3a). Surprisingly, translocation of YAP into the nucleus is observed in local corneal epithelial cells (repairing epithelial cells) near the small wound from 8 to 16 h pw (Fig. 3a, b; Supplementary Fig. 4a). The YAP N/C ratio of repairing epithelial cells increases from 8 to 16 h pw, decreases at 24 h pw, and reverted to unwounded level at 40 h pw when the small wound is fully repaired and re-epithelialized (Fig. 3c; Supplementary Fig. 4b, c). Importantly, YAP activation is restricted in the zone about 700 μm from the leading margin, but does not further extend toward peripheral cornea and limbus (Supplementary Fig. 4a). p-YAP (Ser127) staining shows some repairing epithelial cells with lower fluorescence intensity during corneal epithelial repair (Supplementary Fig. 4d). CTGF staining also shows local upregulation of CTGF restricted in the zone about 700 μm from the leading margin (Supplementary Fig. 4e). Furthermore, the central corneal epithelial defects (1.5 mm diameter) are closed in 24 h, whereas epithelial defects remain about 30% of total defect sizes at 24 h and even aren’t completely closed at 48 h after VTP treatment (Fig. 3d). Taken together, our data suggest that transient activation of YAP locally in repairing epithelial cells mediates corneal epithelial repair after a small wound.

During normal homeostasis, limbal epithelial cells, but not corneal epithelial cells, show highly proliferative activity (limbus about 10%, central cornea about 1%; Supplementary Fig. 4f, g). Repairing epithelial cells exhibit significantly higher proliferative rate (about 7%) when compared with peripheral corneal epithelial cells during small wound repair (Fig. 3e, f). The proliferation of repairing epithelial cells increases from 8 to 16 h pw, decreases at 24 h pw, and reverted to unwounded level at 40 h pw (Supplementary Fig. 4h). In addition, repairing epithelial cells are revealed to be reprogrammed to highly re-express limbal epithelial stem/progenitor cell markers CK14 and p63α (Fig. 3g–j). Importantly, cell proliferation and the expressions of CK14 and p63α are also restricted in the zone about 700 μm from the leading margin (Fig. 3e, g; Supplementary Fig. 4i, j). Furthermore, VTP significantly inhibits cell proliferation and p63α expression of repairing epithelial cells (Fig. 3i–k; Supplementary Fig. 4j). Together, these data suggest that transient activation of YAP locally induces the activation and reprogramming of repairing epithelial cells to re-express limbal epithelial stem/progenitor cell markers and re-enter into cell cycle.

Like the regeneration after a large wound, inhibition of cell proliferation by Mc delays, but not stop, small wound repair (Fig. 3l, m), indicating that there are enough epithelial cells surrounding the wound to migrate to cover the small wound. Few TUNEL+ cells are detected during epithelial repair after small wound again (Supplementary Fig. 4k). Furthermore, VTP further delays wound closure after Mc treatment (Fig. 3l, m), indicating the role of YAP in epithelial migration during repair after a small wound. Although we cannot reveal the origin of these migratory cells, it is obvious that both proliferative repairing epithelial cells and superficial and suprabasal epithelial cells near the wound contribute to the migration, just like the regeneration after a large wound. Taken together, our data demonstrate that transient activation of YAP locally mediates LESCs-independent repair after a small wound through regulating cell proliferation and migration by activating/reprogramming local repairing epithelial cells.

### Loosening of adhesions and disrupted cortical F-actin cytoskeleton during regeneration and repair

**Fig. 4:**
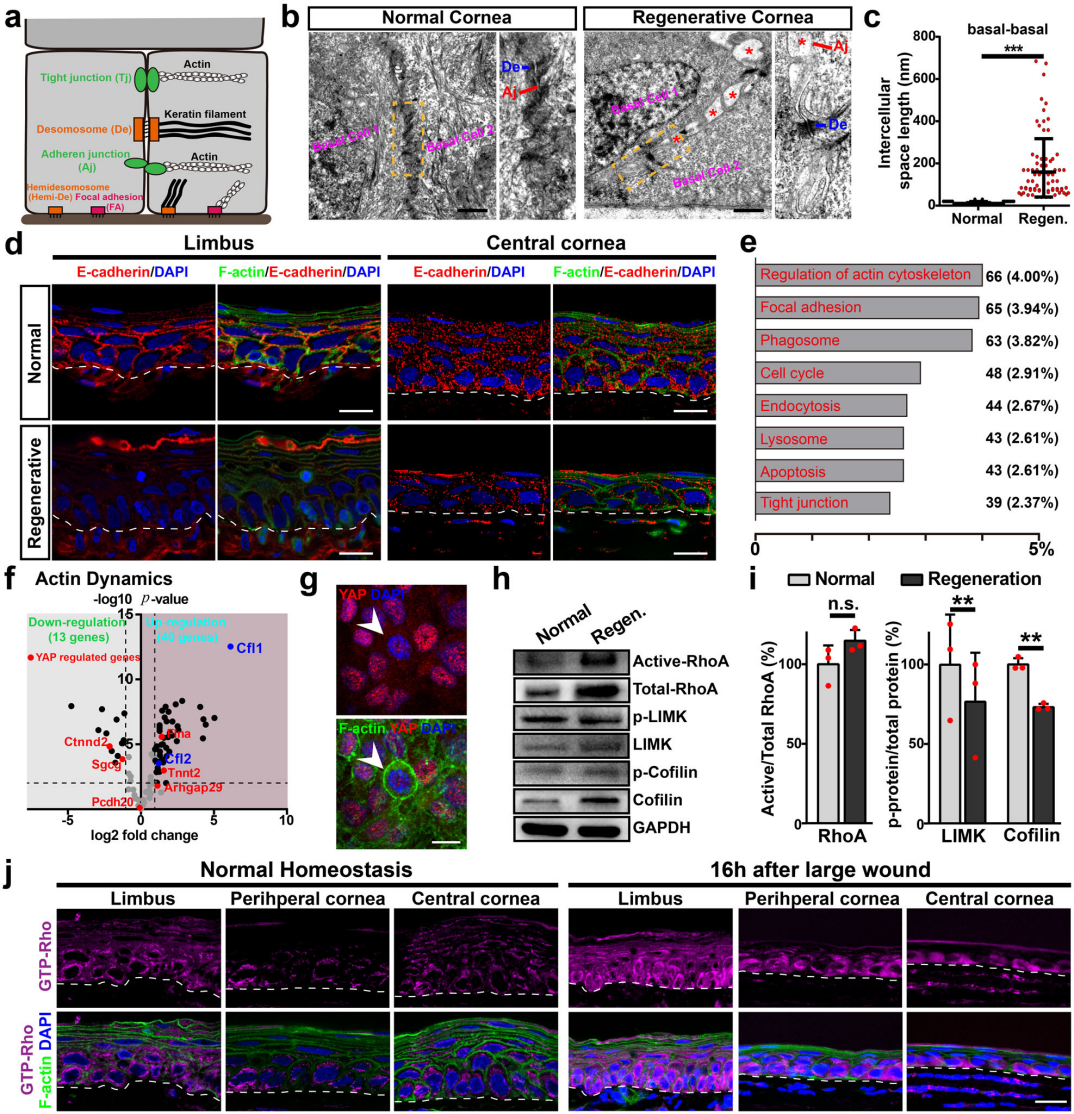
Loosening of adhesions and disrupted cortical F-actin cytoskeleton during regeneration and repair. **a** Schematic representation shows three classes of cell–cell adhesions and two classes of cell-ECM adhesions in the corneal epithelium. **b** TEM images of neighboring epithelial basal cells during normal homeostasis and regeneration at 16 h after large wound. Clear intercellular loosening of Aj at 16 h pw is indicated with asterisks. **c** Quantifications of intercellular space lengths between neighboring epithelial basal cells during normal homeostasis and regeneration (regen.) at 16 h after large wound. **d** E-cadherin and fluorescein-phalloidin stainings of limbal and corneal epithelial cells during normal homeostasis and at 16 h after large wound (regenerative). **e** Enrichment of differential expressed transcripts in KEGG pathways during corneal wound regeneration. The transcript number and percentage are shown. **f** Differentially expressed genes that involving actin dynamics during corneal wound regeneration from published genome-wide cDNA microarray data. YAP target genes (red dots and texts) and Cfl1/2, key proteins that regulate actin dynamics, are shown. **g** YAP and fluorescein-phalloidin stainings of cultured hCECs. Arrows indicate hCECs exhibit low protein level of YAP with the assembly of cortical F-actin cytoskeleton. **h**, **i** Western analysis and quantification of active RhoA/Total RhoA, p-LIMK/LIMK, and p-Cofilin/Cofilin in normal corneal epithelium and regenerative epithelial cells at 16 h after large wound. **j** Active GTP-bound Rho staining and cortical F-actin cytoskeleton staining during normal homeostasis and regeneration at 16 h after large wound. Scale bars, 1 μm (**b**) or 20 μm (**d**, **g**, **j**). Data are the mean ± SD, *n* = 3 experiments (**i**); n.s. not significant, **p < 0.01, ***p < 0.001; Student’s two-tailed unpaired *t*-test (**c**) or paired *t*-test (**i**).

To explore the underlying mechanism of YAP activation in the regeneration and repair, histological features of corneas during homeostasis and regeneration are examined. Transmission electron microscopy (TEM) shows that adherens junction (Aj) and tight junction (Tj), which link unwounded epithelial cells, are disrupted between loosening regenerative epithelial cells, whereas hemidesmosome (Hemi-De) is lost and desmosome (De) is partially retained during regeneration (Fig. 4a, b; Supplementary Fig. 5a–d). The intercellular space length of basal cells-basal cells and basal cells-wing cells ranges from 10 nm to 30 nm in unwounded corneal epithelium, whereas it displays a dramatic increase to about 200 nm at 16 h pw during regeneration (Fig. 4c; Supplementary Fig. 5e). E-cadherin (the central component of Aj) and ZO-1 (the key adaptor within Tj) diminishes in limbal epithelial cells and shows down-regulation in regenerative epithelial cells (Fig. 4d; Supplementary Fig. 5f). Interestingly, phosphorylated focal adhesion kinase (p-FAK, Tyr397), a regulator of focal adhesions, is revealed to be cortical, but not only basal, expression and its fluorescence intensity decreases in limbal epithelial cells (Supplementary Fig. 5g, h). This is consistent with the roles of FAK in regulating epithelial cell–cell contact formation and barrier function^24,25^, as well as the disrupted cell–cell adhesions (Aj and Tj) of limbal epithelial cells during regeneration. Together, these results suggest that these adhesions are disrupted in limbal epithelial cells and regenerative epithelial cells during regeneration.

RNA-seq based on the third generation Nanopore platform enriches cellular processes implicated in the regulation of actin cytoskeleton, focal adhesion, and tight junction during regeneration (Fig. 4e). As Aj and Tj link to F-actin cytoskeleton, we also reveal a large number of genes related to actin cytoskeleton dynamics are differentially regulated during regeneration by analyzing previous genome-wide cDNA microarray data^19^. These differentially expressed genes involve actin cytoskeletal stability and dynamics, connection to the membrane, cell junction (Aj, Tj, and De), focal adhesion and Rho family signaling pathway (Fig. 4f; Supplementary Table 3). Thus, we next investigate the change of cortical F-actin cytoskeleton during regeneration using phalloidin staining. We observe dramatic disruption of cortical F-actin cytoskeleton in limbal and regenerative epithelial cells during regeneration (Fig. 4d, j; Supplementary Fig. 5f). Similarly, the disruption of cortical F-actin cytoskeleton is observed in repairing epithelial cells at 8 and 16 h after a small wound, and this disruption is reverted back to that seen in unwounded epithelium when the small wound is completely repaired and re-epithelialized (Supplementary Fig. 5i). Thus, these data suggest that adhesions/junctions and cortical F-actin cytoskeleton are disrupted during corneal epithelial regeneration and repair.

### Manipulating F-actin cytoskeleton regulates YAP activity

The common YAP activation during LESCs-dependent regeneration and LESCs-independent repair implies that a common molecular mechanism that activates YAP might also be shared. The cortical F-actin cytoskeleton, which links to cell junctions/adhesions, is a potential common sensor that relays signals from the disrupted adhesions and tissue integrity to affect YAP activity. The correlation between the disrupted cortical F-actin cytoskeleton and YAP activation during regeneration and repair indicates that cortical F-actin cytoskeleton might suppress YAP nuclear localization and its activity, or vice versa. In support of this view, cultured hCECs with cortical F-actin cytoskeleton display low expression and inactivation (predominantly cytoplasmic) of YAP, whereas hCECs that do not assemble cortical F-actin cytoskeleton show high expression and activation (predominantly nuclear) of YAP (Fig. 4g).

Rho/ROCK signaling pathway played important roles in epithelial organization and dynamics of cortical F-actin cytoskeleton, cell proliferation, differentiation, and adhesion through myosin light chain (MLC) and/or LIMK1/2-Cofilin. Cofilin, a potent actin depolymerizing factor and regulator of actin dynamics, is regulated by LIMK1/2 through Ser3 phosphorylation, which blocks Cofilin activity and results in the stabilization of F-actin^26^. Compared with normal homeostasis, the protein level of Cofilin is upregulated, and the p-Cofilin/Cofilin ratio significantly decreases during regeneration (Fig. 4h, i), indicating enhanced Cofilin activity and actin dynamics. In agreement with the enhanced Cofilin activity, the p-LIMK/LIMK ratio significantly decreases, suggesting the inhibition of ROCK/LIMK/Cofilin pathway during regeneration. However, the protein level of GTP-bound active RhoA and the ratio between GTP-bound active RhoA and total RhoA increase (Fig. 4h–j), which is consistent with previous data that RhoA and ROCK exert opposite effects on wound healing in vitro^27,28^. Together, these results demonstrate that inhibition of ROCK/LIMK/Cofilin pathway enhances Cofilin activity and accelerates actin dynamics, which results in the instabilization and disruption of cortical F-actin, during regeneration.

**Fig. 5:**
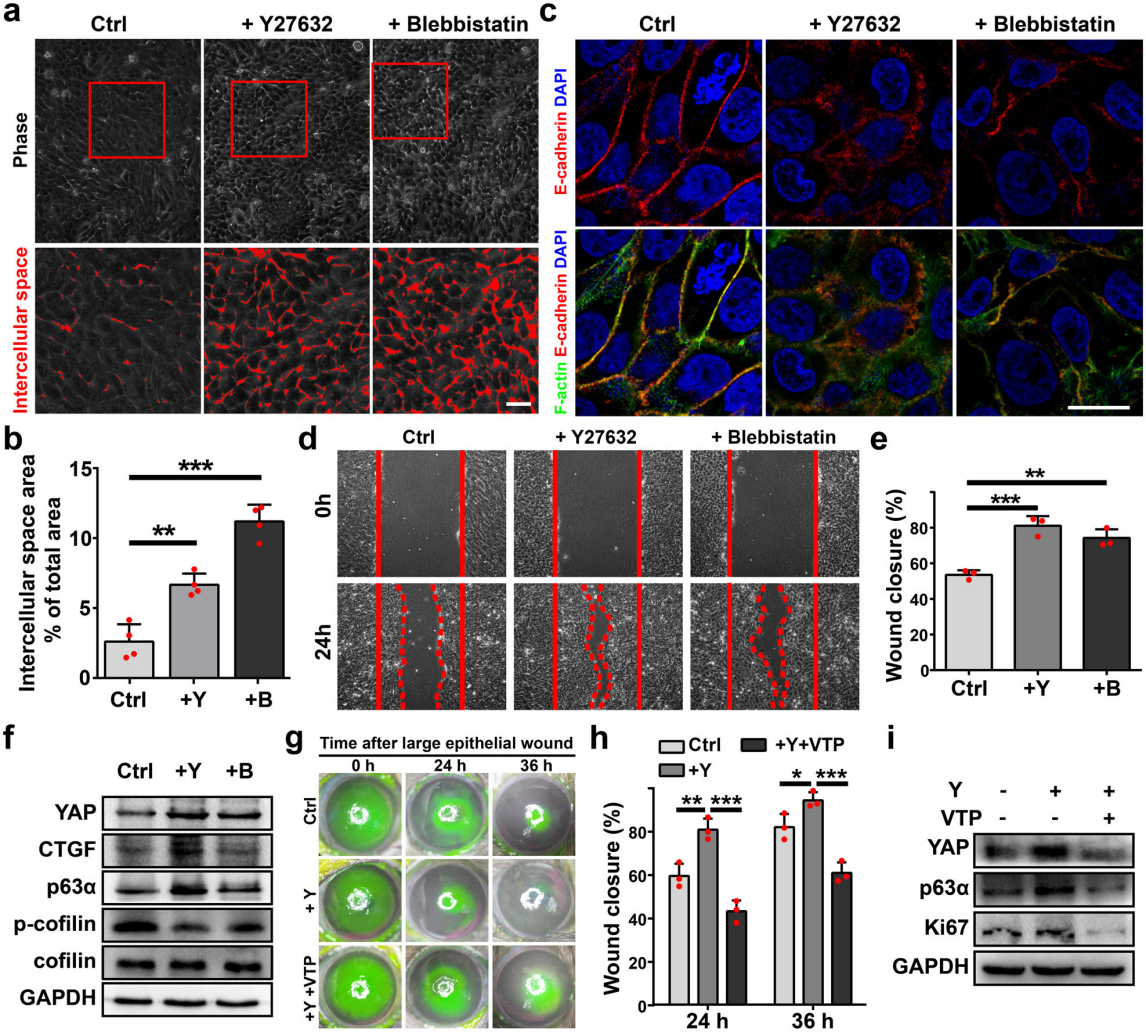
Manipulating F-actin cytoskeleton regulates YAP activity. **a**, **b** Intercellular spaces between hCECs under indicated treatments for 8 h, and quantifications of intercellular spaces between hCECs. **c** E-cadherin and fluorescein-phalloidin stainings of hCECs under indicated treatments for 8 h. **d**, **e** hCECs cells are scratch wounded at confluence, and images are captured at 0 and 24 h post-scratching. Quantifications of wound closure (%) at 24 h post-scratching. **f** Western analysis of indicated proteins from hCECs cells under indicated treatments for 24 h. **g**, **h** The corneal epithelial defects are stained with fluorescein sodium at 0, 24, and 36 h after 4 mm-diameter wound under indicated conditions. Quantifications of wound closure (%) at 24 and 36 h. **i** Western blotting analysis of indicated proteins from rabbit primary cultured LESCs cells under indicated treatments for 24 h. Scale bars, 20 μm (**a**, **c**). Data are the mean ± SD, *n* = 4 biological replicates (**b**), *n* = 3 biological replicates (**e**), *n* = 3 corneas (**h**); *p < 0.05, **p < 0.01, ***p < 0.001; One-way ANOVA with Dunnett’s post test (**b**, **e**) or Student’s two-tailed unpair**e**d *t*-test (**h**). Ctrl, 0.1% DMSO; Y27632, 20 μM (**a**–**c**) or 10 μM (**d**–**i**); Blebbistatin, 30 μM (**a**–**c**) or 20 μM (**d**–**f**); VTP, 10 μM (**g**, **h**) or 5 μM (**i**).

To further explore whether the cortical F-actin cytoskeleton regulates YAP activity and affects corneal epithelial regeneration/repair, we manipulate the ROCK pathway using specific inhibitors. Y-27632, an inhibitor of ROCK, and blebbistatin, an inhibitor of myosin II, significantly increase intercellular spaces between hCECs (Fig. 5a, b), disrupt Aj, Tj, and cortical F-actin cytoskeleton (Fig. 5c; Supplementary Fig. 6a), and accelerate wound closure through promoting cell proliferation in vitro (Fig. 5d, e; Supplementary Fig. 6b-d). Importantly, Y-27632 and blebbistatin increase YAP protein level and nuclear localization, and upregulate YAP target genes, e.g., Areg, Ereg, and CTGF (Fig. 5f; Supplementary Fig. 6e–g). However, Y-27632 and blebbistatin do not upregulate the mRNA level of YAP (Supplementary Fig. 6e), indicating that Y-27632 and blebbistatin regulate YAP activation through post-transcriptional mechanism. In addition, the accelerated wound closure by Y27632 treatment is delayed by VTP in vitro (Supplementary Fig. 6h, i).

Furthermore, Y-27632 accelerates corneal epithelial wound healing in vivo, and VTP inhibits the accelerated wound healing by Y27632 (Fig. 5g, h). The increased expressions of proliferative marker Ki67 and LESCs marker p63α after Y27632 treatment indicate that Y27632 promotes proliferation and stem cell pool expansion (Fig. 5i). Collectively, all these data demonstrate that disrupted cortical F-actin cytoskeleton and enhanced actin dynamics by ROCK/LIMK/Cofilin inhibition relieve the suppression of cortical F-actin cytoskeleton on YAP activity, and accelerate corneal epithelial regeneration and repair.

### YAP as a key regulator of the assembly of cell junction and cortical F-actin cytoskeleton

Previously, YAP chromatin immunoprecipitation followed by deep sequencing (ChIP-Seq) analysis revealed specific DNA-binding signature for genes involved in cytoskeleton stabilization and connection to the membrane^29^. Remarkably, potential target genes of YAP that implicate in actin cytoskeletal stability and dynamics (e.g., Sgcg, Flna, and Tnnt2), connection to the membrane and cell junction (e.g., Ctnnd2), focal adhesion (e.g., Tln2, Zyx, Itga, and Itgb) and Rho family signaling pathway (e.g., Arhgap29) are differentially expressed during corneal epithelial regeneration (Fig. 4f; Supplementary Table 3). This implies that YAP might, in turn, suppress the assembly of cell junction and cortical F-actin cytoskeleton via Rho/ROCK signaling pathway, forming a feedback-regulatory loop.

**Fig. 6:**
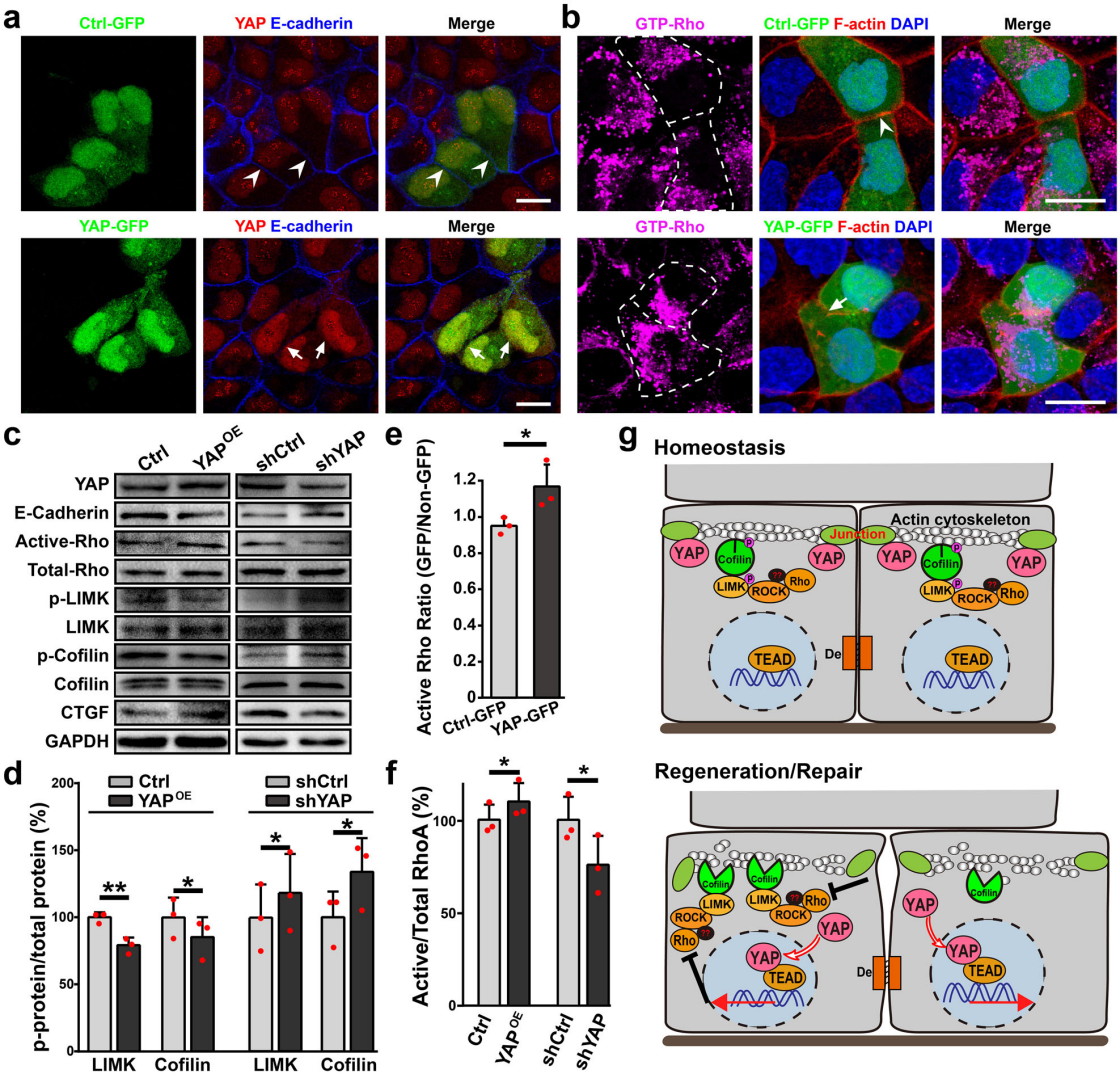
YAP as a key regulator of the assembly of cell junction and cortical F-actin cytoskeleton. **a** YAP-overexpressing hCECs (YAP-GFP) and hCECs infected with control lentivirus (Ctrl-GFP) are co-cultured with uninfected hCECs (without GFP). YAP and E-cadherin stainings for hCECs. Arrows and arrowheads indicate present or absent E-cadherin staining, respectively, at the interface between two infected hCECs. **b** Staining of hCECs for active GTP-bound Rho and cortical actin cytoskeleton. The broken white lines show GFP-positive infected hCECs. Arrows and arrowheads indicate present or disrupted cortical F-actin cytoskeleton, respectively, at the interface between two infected hCECs. **c** Control, YAP-overexpressing hCECs (YAP-OE), shCtrl and YAP-knockdown hCECs (shYAP) are immunoblotted to evaluate RhoA activity, expressions of YAP, E-cadherin, LIMK and Cofilin, and phosphorylation of LIMK and Cofilin. **d** Quantification of p-LIMK/LIMK and p-Cofilin/Cofilin in control hCECs and YAP-overexpressing hCECs (YAP-OE) or YAP-knockdown hCECs (shYAP). **e** The GTP-bound active Rho ratio of immunofluorescence intensity between GFP-positive infected hCECs (Ctrl or YAP-overexpressing) and GFP-negative uninfected hCECs. **f** Quantification of active GTP-bound RhoA/Total RhoA in control hCECs and YAP-overexpressing hCECs (YAP-OE) or YAP-knockdown hCECs (shYAP). **g** Schematic representations of the reciprocal regulation of YAP activity and the assembly of cell junction and cortical F-actin cytoskeleton via the ROCK/LIMK/Cofilin pathway. Scale bars, 20 μm (**a**, **b**). Data are the mean ± SD, *n* = 3 experiments (**d**, **f**), *n* = 3 fields (**e**); *p < 0.05, **p < 0.01; Student’s two-tailed paired *t*-test (**d**, **f**) or unpaired *t*-test (**e**).

To evaluate this hypothesis, we overexpress or knockdown YAP in hCECs using lentivirus and co-culture these YAP-overexpressing or -knockdown hCECs (with GFP) with non-infected hCECs (without GFP). As a control, hCECs infected with control lentivirus (only expressing GFP) are co-cultured with uninfected hCECs. We observe enhanced phase brightness surrounding YAP-overexpressing hCECs when compared with hCECs infected with control lentivirus and uninfected hCECs (Supplementary Fig. 7a, c, d), indicating reduced intercellular adhesion. Interestingly, some cells neighboring YAP-overexpressing hCECs also show enhanced phase brightness (Supplementary Fig. 7a). Consistent with the reduced intercellular adhesion to neighboring cells, YAP-overexpressing hCECs exhibit reduced E-cadherin between cell contact sites, whereas hCECs infected with control lentivirus and uninfected hCECs exhibit normal E-cadherin junctional boundaries (Fig. 6a). Similar results are revealed using Tj adaptor protein ZO-1 (Supplementary Fig. 7f). In contrast, YAP-knockdown hCECs show decreased phase brightness when compared with hCECs infected with control lentivirus and uninfected hCECs (Supplementary Fig. 7b, c, e), and exhibit normal E-cadherin and ZO-1 junctional boundaries (Supplementary Fig. 7f, g). In addition, YAP-overexpressing hCECs show decreased E-cadherin protein level, whereas YAP-knockdown hCECs show increased E-cadherin protein level when compared with hCECs infected with control lentivirus (Fig. 6c). Together, these results suggest that YAP regulates the assembly of cell junctions.

Furthermore, hCECs infected with control lentivirus and uninfected hCECs exhibit clear cortical F-actin, whereas cortical F-actin between YAP-overexpressing hCECs is disrupted (Fig. 6b). The GTP-bound active Rho ratio of immunofluorescence intensity between GFP-positive hCECs infected with control lentivirus and GFP-negative uninfected hCECs is about 1.0, indicating that control lentivirus infection has no effect on Rho activity. The GTP-bound active Rho ratio between GFP-positive YAP-overexpressing hCECs and GFP-negative uninfected hCECs significantly increases (Fig. 6b, e). Western blotting also reveals increased GTP-bound active RhoA level and active/total RhoA ratio, suggesting enhanced Rho activity in YAP-overexpressing hCECs. However, p-LIMK/LIMK ratio and p-Cofilin/Cofilin ratio decrease in YAP-overexpressing hCECs when compared with hCECs infected with control lentivirus (Fig. 6c, d, f).

In contrast, although the GTP-bound active Rho ratio of immunofluorescence intensity between YAP-knockdown hCECs and uninfected hCECs doesn’t significantly change (Supplementary Fig. 7h, i), Western blotting reveals decreased GTP-bound active RhoA level and active/total RhoA ratio. However, p-LIMK/LIMK ratio and p-Cofilin/Cofilin ratio increase in YAP-knockdown hCECs when compared with hCECs infected with control lentivirus (Fig. 6c, d, f). Thus, these results suggest that YAP regulates the ROCK/LIMK/Cofilin signaling pathway and the assembly of cortical F-actin cytoskeleton in corneal epithelial cells. Collectively, all these results demonstrate that disrupted cortical F-actin cytoskeleton and enhanced actin dynamics by inhibition of ROCK/LIMK/Cofilin pathway relieve the suppression on YAP activity. In turn, YAP regulates the assembly of cell junction and cortical F-actin cytoskeleton of epithelial cells, indicating the formation of a regulatory loop (Fig. 6g).

## Discussion

Adult tissues must maintain themselves and activate regenerative/repairing responses to restore their functions after injury. Unfortunately, most adult organs in humans have limited or no potential to regenerate/repair properly patterned and functional tissues after injury. Instead, injury often triggers scaring and fibrosis that ultimately lead to organ malfunction^30^. In general, functional regeneration/repair of a tissue is endowed by the resident ASCs, such as in muscle, skin, intestine, and corneal epithelium (after a large wound). However, recent studies uncover many examples across organs where cells that are fate/lineage restricted and even terminally differentiated can re-enter stem-cell-like state or be re-activated to proliferate to regenerate/repair injured tissues. For example, regenerated hepatocytes are derived from proliferation of pre-existing fully differentiated hepatocytes without dedifferentiation and reprogramming^1,2^. Are these regeneration/repair mediated by as-yet-unknown ASCs or by differentiated cells according to organs’ needs? It remains a matter of continued debate in regenerative biology. Here, we provide another example of regeneration/repair after injury in corneal epithelium, where lineage restricted and differentiated cells are called to cope with the small wound even when the ASCs exist.

According to the LESC hypothesis, LESCs act as the source of new basal corneal epithelial cells during normal homeostasis and become more active during regeneration after large wound. However, a small wound could be healed without the activation of LESCs. This LESCs hypothesis has been challenged by an alternative corneal epithelial stem cell (CESC) hypothesis, which proposes that corneal epithelial homeostasis is maintained by stem cells scattered throughout the corneal epithelium^31^. However, recent lineage tracing experiments argue against the CESC hypothesis and suggest that the centrally located K14+ CESCs exist only in early postnatal life, which are progressively replaced by LESCs^32–34^. It is thought that corneal epithelial injuries are resolved by leading-edge cells “sliding”/“rolling” into the wound bed or by “basal cell migration”, where CK14+ basal epithelial cells are forced into the wound bed by increased population pressure^23^. In this study, we show that large-sized wound and small-sized wound are dealt with by LESCs and reprogrammed local repairing epithelial cells, respectively. Thus, corneal epithelial response to injury is correlated to the wound size, and the regeneration after a large wound and the repair after a small wound should be considered as two different processes, even if they share some common mechanisms. Recently, differentiated/committed corneal epithelial cells were reported to migrate back to the intact limbal niche and dedifferentiate into stem cells following surgical deletion of the LESCs^35^. Herein, we show that repairing epithelial cells re-express limbal epithelial stem/progenitor cell markers CK14 and p63α, and inhibition of YAP significantly inhibits the expression of p63α in repairing epithelial cells. Previously, YAP activation was reported to contribute to the fate changes of corneal epithelial cells after topical applications of type-I collagenase and under chronic inflammation^36,37^. Whether these repairing epithelial cells are indeed dedifferentiated into stem cells functionally independent of the limbal niche and whether YAP contributes to the dedifferentiation need further studies.

During regeneration/repair, the common process is to replace the injured tissues/cells with the production of new cells. A variety of routes and mechanisms are used to produce new cells, and it varies across tissues, such as proliferation of resident ASCs (e.g., in muscle and intestine) or terminally differentiated cells (e.g., in liver), dedifferentiation of mature cells into a stem cell-like state (e.g., in lung alveolus and intestine crypt), and even trans-differentiation into another cell type^38^. No matter which route is used, the early signaling events after injury must be the activation of surrounding cells to produce new cells through proliferation. Thus, a common mechanism might be shared by ASCs-dependent and ASCs-independent regeneration/repair to re-activate the proliferation. Herein, we demonstrate the Hippo/YAP pathway to be such a common mechanism that are shared by LESCs and differentiated cells to re-activate the proliferation and heal corneal epithelial wound with different sizes. The highly conserved Hippo/YAP pathway, first widely investigated in contact inhibition of growth^39^, is regulated by a diverse array of upstream signals and cross-talks with other signaling pathways. The complex regulations make Hippo/YAP pathway evolve to be a key common mechanism that couples tissue architecture to growth control, where YAP/TAZ sense and integrate signals to control not only cell survival and proliferation, but also cellular plasticity and fate determination and expansion of stem/progenitor cell compartments that are essential for tissue regeneration/repair^13,15,16^. Consequently, Hippo/YAP pathway becomes exquisitely sensitive to perturbation of normal tissue and cellular integrity, e.g., cell polarity, cell junctions, cell tension, and so on. This ensures that any disruption of the normal tissue and cellular architecture can be effectively restored by compensatory cell proliferation mediated by YAP/TAZ^15,16^. Therefore, it is not surprising that Hippo/YAP pathway commonly contributes to regeneration/repair in intestine, liver, lung, muscle, and even in heart, irrespective of the tissues/organs contain ASCs or not^15^. Thus, the manipulation of YAP activation provides potential therapeutic approach to harness the plasticity of differentiated cells for in situ tissue regeneration/repair.

Epithelial cells mainly function to establish a barrier that protects underlying tissues and maintain physiological environment. Cell–cell junctions (Aj and Tj) form extracellular connections between adjacent cells and intracellular links to cortical F-actin cytoskeleton, which integrate epithelial cells to be a structural continuum across the tissue. This makes cell junction and cortical F-actin cytoskeleton to be a sensor for tissue integrity, which could rapidly respond to any perturbations of tissue homeostasis. In addition to be structural organization, cell junction and cortical F-actin cytoskeleton also organize large signaling networks at these junctional sites, such as Rho family (RhoA, Rac, and CDC42), Wnt/β-catenin and Hippo/YAP signaling pathways, to regulate cell proliferation, migration and wound healing^40^. The compensatory proliferation occurs either by exposure to new signals or by release from inhibitory signals. Obviously, the activity of YAP is suppressed by normal tissue and cellular integrity. Thus, once tissue and cellular integrity are disrupted, inhibitory signals are eliminated and YAP rapidly and effectively restores the injured tissue. Here, we demonstrate that the assembly of cell junction and cortical F-actin cytoskeleton function as an inhibitory signals of YAP activity in corneal epithelium, and the reciprocal regulation of YAP activity and the assembly of cell junction and cortical F-actin cytoskeleton forms a double-negative feedback-regulating loop. The mutual inhibition between Rac1 and RhoA results in a bistable activity system, and the bistable properties of the Rac1-RhoA biochemical circuitry are translated into bistability of the actin dynamics and cell migration^41^. Thus, the double-negative feedback loop resulting from mutual inhibition between the assembly of cell junction and cortical F-actin cytoskeleton and YAP activity may also lead to a bistable activity system, just like Rac1 and RhoA. In addition, the assembly of cell junction and cortical F-actin cytoskeleton and the RhoA/ROCK pathway forms a positive-feedback loop^42^. Thus, it seems that epithelia are organized to be signaling networks with a variety of regulatory-loops and feedbacks, especially these bistable activity systems, which make it sensitive for any perturbations and enable a switch-like, abrupt and digital response to cope with the injury. In addition, our data indicate that RhoA and ROCK might exert different effects on corneal epithelial wound healing in vivo, consistent with previous results in vitro^27,28^. Similar phenomenon was revealed in the culture of human embryonic stem cells (hESCs), where inhibition of Rho or ROCK showed opposite effects on the survival of hESCs and actin dynamics and YAP activity mediated this difference^43^. We speculate that the different spatial distribution of modulators (including GEFs, GAPs, and GDIs) and different downstream effectors (including mDia and ROCK) of Rho might result in the opposite roles of RhoA and ROCK in the wound healing, which needs further studies.

## Methods

### Corneal epithelial debridement wound regeneration/repair and drug treatments

Adult Long Evans rats and New Zealand white rabbits used in the present study were housed in a controlled environment with standard conditions of temperature and humidity with a cycle of an alternating 12 h light and dark. These animal protocols were approved by the Laboratory Animal Welfare and Ethics Committee of the Third Military Medical University (Army Medical University), Chongqing, China. Four-month-old Long Evans rats were anesthetized by mixed O_2_ and isoflurane (RWD Life Science). After application of two drops of topical proparacaine (0.5%), the central cornea was marked by a trephine with 4 mm or 1.5 mm in diameter and corneal epithelium was peeled off using forceps under a dissecting microscope. To quantify the wound regeneration or repair, defects of corneal epithelium were visualized by instilling 0.25% fluorescein sodium at indicated time. The area of defect was quantified using ImageJ software and the wound closure was calculated as the percentage of the healed epithelial area/initial wound area at indicated time point. For drug treatments, vehicle (0.1% DMSO; or H_2_O), Y-27632 (10 μM, Peprotech), or verteporfin (VTP, 10 μM, MedChem Express), and mitomycin C (Mc, 5 μg/mL; Selleck) were pre-treated for 1 h and administrated on cornea every 8 h after the scrape wound. These administrated drugs were maintained on the corneas of anesthetized rats for at least 1 h. All experiments where VTP was used were performed in dark to protect from direct sunlight.

### Cell cultures

Rabbit primary limbal epithelial stem cells (LESCs) were cultured from New Zealand white rabbits (4–5 months) limbal tissue explants using a previous protocol with modification^44^. In brief, an incision was made at the conjunctiva of the eye 1 mm behind limbus and dissected toward limbus and into the cornea up to 1 mm. After the conjunctiva was excised out at the limbus, the limbal ring tissue was cut into 18-20 pieces with 1-2mm^2^. These pieces were directly put onto 6-well culture plates with epithelium side up. After incubation in a drop of fetal bovine serum (FBS, Hyclone) for 16 h, limbal tissue explants were grown in DMEM/F12 (1:1) medium (Hyclone), supplemented with 10% FBS, insulin-transferrin-selenium (Gibco), and 1% (v/v) penicillin-streptomycin (Hyclone). The media was changed every 2 days. The LESCs of passage 1 (P1) to P3 were used in this study.

Human corneal epithelial cells (hCECs) were obtained from BeNa Cuture Collection (Beijing, China) and maintained in DMEM/F12 (1:1) medium supplemented with 6% (v/v) FBS (Hyclone) and 1% (v/v) penicillin/streptomycin. The hCECs of P5 to P10 were used in this study. All the cells were cultured at 37 °C in cell culture incubator with humidified environment in 5% CO_2_.

### Lentivirus infection and co-cultures

To generate YAP-knockdown hCECs, hCECs cells were infected with lentivirus containing shRNA-targeting YAP1 with EGFP expression (GV115-shYAP1; GENE, Shanghai, China) or control lentivirus (GV115 empty vector) with EGFP expression (GENE, Shanghai, China). To generate YAP-overexpressing hCECs, hCECs cells were infected with lentivirus containing human YAP1 cDNA (NM_001130145) with EGFP expression (GV358-YAP1; GENE, Shanghai, China) or control lentivirus (GV358 empty vector) with EGFP expression (GENE, Shanghai, China). The MOI (multiplicity of infection) was 30, and the infectious medium (DMEM/F12 with 2% FBS and 4% infectious reagent) was changed after 12 h. hCECs cells were further cultured for 60 h and followed by co-culture or Western blotting assays.

For co-cultures, hCECs cells infected with lentivirus (YAP-knockdown, YAP-overexpression, or control lentivirus) and uninfected hCECs cells were co-seeded with the ratio 1:10. Alternatively, hCECs cells in low density were infected with lentivirus for 12 h, and the infectious medium was changed and uninfected hCECs cells were seeded in high density. The co-cultured hCECs cells were cultured for 48 h and followed by subsequent immunofluorescence staining.

### Immunohistochemistry, immunofluorescence staining

Rat corneal tissues were fixed in 4% paraformaldehyde (PFA), incubated in 30% sucrose and embedded in Tissue-Tek OCT compound. Frozen sections of tissues (10μm thick sections) were cut using a Leica cryostat, and mounted on microscope slides. Cultured cells were fixed in 4% PFA. After permeabilized in 0.3% Triton X-100 for 10 min, blocked in 3% bovine serum albumin (BSA) for 60 min, corneal sections or cultured epithelial cells were incubated with primary antibodies at 4 °C for 16 h. After washes with PBS and subsequently with fluorescein-conjugated secondary antibodies at 37 °C for 1 h, all samples were washed with PBS and counterstained with DAPI. After immersed in anti-fade medium and mounted on glass slides, all samples were examined using a fluorescence microscope (Olympus BX51) or confocal microscope (Leica SP5/8; Zeiss LSM800). The following primary antibodies were used: rabbit anti-YAP1 polyclonal antibody (Abcam, ab39361; 1:400), rabbit anti-YAP1 monoclonal antibody (Abcam, ab205270; 1:300), rabbit p-YAP (Ser127; Abcam, ab76252; 1:200), rabbit anti-Ki67 polyclonal antibody (Abcam, ab15580; 1:400), rabbit anti-Ki67 polyclonal antibody (Milipore, AB9620; 1:500), mouse anti-pH3 (phospho-Histone H3 (Ser10)) monoclonal antibody (Milipore, 05-806; 1:130), rabbit anti-p63α monoclonal antibody (Abcam, ab124762; 1:800), rabbit anti-CK14 monoclonal antibody (Abcam, ab181595; 1:200), rabbit anti-CK12 monoclonal antibody (Abcam, ab185627; 1:150); mouse anti-CTGF monoclonal antibody (Santa Cruz, sc-365970; 1:600), mouse anti-E-cadherin monoclonal antibody (Abcam, ab1416; 1:50), rabbit anti-ZO-1 polyclonal antibody (Invitrogen, 40-2200; 1:200), rabbit anti-p-FAK monoclonal antibody (Tyr397; Abcam, ab81298; 1:200) and rabbit anti-FAK monoclonal antibody (Abcam, ab40794;1:300). The following secondary antibodies were used: Goat anti-mouse Alexa-Fluor-647 (Life technologies, A21236; 1:800), Goat anti-Rabbit Alexa-Fluor-647 (Life technologies, A32733; 1:800), Goat anti-rabbit Alexa-Fluor-568 (Life technologies, A11011; 1:500), Donky anti-mouse Alexa-Fluor-568 (Life technologies, A10037; 1:500), Goat anti-rabbit Alexa-Fluor-488 (Life technologies, A11001; 1:400) and Goat anti-mouse Alexa-Fluor-488 (Life technologies, A32723; 1:400).

For phalloidin staining, corneal sections or cultured cells were incubated in fluorescein-phalloidin (Sigma, P5282; Invitrogen, A12380; 1:800) for 10 min following the immunofluorescence staining. For quantification of the percentage of indicated markers (Ki67, p63, and CK14), 200–300 μm epithelial cells were counted.

For YAP subcellular distribution, cellular borders were identified with phalloidin staining, and nuclei were segmented using the DAPI signal. The immunofluorescence intensity of nuclear and cytoplasmic YAP was quantified, and the ratio between nuclear and cytoplasmic YAP (N/C ratio) was calculated. The three categories, nuclear (N), cytoplasmic (C), and nuclear and cytoplasmic (N/C), were defined based on the N/C ratio as shown in Fig. 1a.

### RT-qPCR

Total RNA was extracted from rat corneal epithelium (normal or wounded at 16 h after large wound) or hCECs cells after indicated treatments using RNAiso Plus (TaKaRa, 9109) according to the manufacture’s protocol. The mRNA quality (260/280 ratio) was assessed using a Nanodrop 2000 spectrophotometer (Thermo Scientific), and was ensured to be within the 1.8–2.0 range for further assay. DNase-treated RNA was reversely transcribed into cDNA using PrimeScript RT reagent Kit (TaKaRa) with the following parameters: 15 min at 37 °C followed by 5 s at 85 °C. Real-time qPCR was performed using cDNA (100 ng/reaction), SYBR Green Premix Ex Taq (TaKaRa) or SYBR Green 2× qRCR Master Mix (Bimake) and primers of genes on Bio-Rad CFX96 Real-Time System with the following parameters: 40× two-step cycle: denaturation, 5 s at 95 °C; annealing and elongation, 40 s at 60 °C. Relative gene expression was quantified using the delta-delta Ct method. Relative expression levels of the genes of interest were normalized to GAPDH as an endogenous control. Data were represented as gene expression relative to control group from 3 biological replicates. The sequences of the primers are listed in Supplementary Table 4.

### Western blotting analysis

Total protein was extracted from the lysed samples in RIPA buffer with PMSF (Beyotime) on ice. After centrifuged at 10,000 rpm at 4 °C for 20 min, the supernatant was collected. Protein concentration was determined using the BCA assay method. For Western blotting analysis, protein samples were denatured in loading buffer at 95 °C for 10 min. Equal amount of protein was loaded into each well and separated by 8–20% w/v SDS-PAGE gels, then electrophoresed and electrophoretic transferred onto methanol-activated polyvinylidene difluoride (PVDF) membrane (Millipore). The PVDF membranes were blocked with 5% BSA dissolved in TBST (20 mM Tris, 145 mM NaCl, pH 7.6 and 0.02% v/v Tween 20) for 2 h. Primary antibodies were diluted using 5% BSA in TBST and incubated with the PVDF membrane for 16 h at 4 °C. After washes with TBST (three times for 15 min), the target proteins were recognized by proper secondary horseradish peroxidase (HRP)-conjugated antibodies (goat anti-rabbit/mouse; Beyotime, A0208, A0216; 1:3000) for 2 h at room temperature. The immuno-reactive bands were presented by reacting with chemiluminescence reagents (ECL, Beyotime) and the densitometry of the immuno-reactive bands was quantified with ImageJ software using GAPDH as internal control. The primary antibodies include: rabbit YAP1 (Abcam, ab39361; 1:2000), mouse YAP (Santa Cruz, sc-271134; 1:2000), rabbit p-YAP (Ser127; Abcam, ab76252; 1:2000), mouse CTGF (Santa Cruz, sc-365970; 1:2000), rabbit p63α (Abcam, ab124762; 1:2000), rabbit CK14 (Abcam, ab181595; 1:2000), mouse CK15 (Abcam, ab80522; 1:3000), rabbit Ki67 (Abcam, ab15580; 1:800), rabbit p-Cofilin (Ser3; Abcam, ab12866; 1:2000), rabbit Cofilin (Abcam, ab42824; 1:2500), rabbit p-LIMK (LIMK1 Thr 508, LIMK2 Thr 505; CST, 3841S; 1:1000), rabbit LIMK (CST, 3842S; 1:1500), mouse RhoA (Santa Cruz, sc-418; 1:600), mouse E-cadherin (Abcam, ab1416; 1:150); mouse β-actin (Abcam, ab8226; 1:3000) and GAPDH (CWBIO, CW0100M; 1:3000). All blots were derived from the same experiment and processed in parallel. Uncropped Western blotting images were provided in Supplementary Fig. 8, where the size markers were labeled.

### Active Rho pull-down assay and staining

For Rho pull-down assay, cells were lysed in RIPA buffer (Beyotime, P0013D) for 20 min on ice, centrifuged at 10,000 rpm for 5 min at 4 °C and the supernatant was collected. Protein concentration was measured by BCA assay method. An equal amount of protein was used for normal and regenerative epithelial samples. 20% of cell lysis was kept as loading and total Rho controls. The cell lysis was incubated with glutathione agarose beads (Santa Cruz, sc-2009) pre-coated with the Rho-GTP binding domain of human Rhotekin protein tagged with GST (GST-RBD protein; Cytoskeleton Inc., RT01), at 4 °C for 90 min. After washes three times with chilled PBS buffer, the glutathione agarose beads that enriched active GTP-Rho were boiled in loading buffer at 95 °C for 10 min and analyzed by Western blotting using RhoA primary antibody (Santa Cruz, sc-418).

For active Rho staining, corneal sections or hCECs were permeabilized in 0.3% Triton X-100 for 10 min, and blocked in 3% BSA for 60 min. After incubated with 2 μg GST-RBD protein at 4 °C for 16 h, corneal sections or hCECs were followed by PBS washes and incubation with Alexa Fluor 488/647-conjugated anti-GST antibodies (Invitrogen, A11131 and MA4-004-A647; 1:25) at 37 °C for 60 min. All samples were captured using confocal microscope, and the filter settings, gain, and exposure values were kept constant between experiments. For quantification, the fluorescence intensity was determined by manually drawing the region that covered a single hCEC. The fluorescence intensity was the mean intensity multiplied by the cellular area. The GTP-bound active Rho ratio between GFP-positive hCECs and GFP-negative uninfected hCECs from the same field was calculated and plotted with GraphPad Prism software (Version 6.0).

### Transmission electron microscopy (TEM)

Normal corneas and regenerative corneas (16 h post large wound) were fixed in 2.5% glutaric dialdehyde/0.1 M cacodylate buffer for 16 h followed by 1% Osmium Tetroxide fix for 2 h. After dehydrated through an acetone series and soaked in acetone/resin (1:1) for 4 h, corneas were embedded in resin. Blocks were trimmed, and semi-thin sections (1-μm) were counterstained with lead and 3% uranyl acetate and imaged on a JEM-1400Plus transmission electron microcope. The intercellular length was determined using ImageJ.

### In vitro intercellular space analysis

hCECs were plated and grew to a confluent monolayer. After treatment with DMSO (Ctrl), Y27632 or Blebbstatin, hCECs were imaged by LEICA DM IRE2 light microscope (LEICA). To quantify the intercellular space, the extent of phase-bright spaces between neighboring epithelial cells was determined using threshold analysis in ImageJ. The threshold value was set at 160. Percentage intercellular space was calculated as a function of phase-bright intercellular space area/the total measured area using ImageJ.

### Terminal deoxynucleotidyl transferase dUTP nick end labeling (TUNEL) staining

TUNEL staining was performed using In Situ Cell Death Detection Kit, TMR red (Roche, 12156792910) according to the manufacture’s protocol, and samples were counterstained with DAPI staining.

### In vitro scratch wound healing assay

hCECs were plated on 6-well culture plates in culture medium and grew to a confluent monolayer. hCECs were pre-treated under indicated conditions, then hCECs were scratch wounded using a sterile 200 μL pipette tip with three linear scratches across the center of the well. After washed three times with PBS to remove cell debris, hCECs were cultured under indicated conditions. Marked lines back the culture plates were made as a reference for camera positioning. Immediately after the scratch and at indicated time point after scratch wound, images of the same field of view were taken using the LEICA DM IRE2 light microscope (LEICA). The remaining wound area was quantified with ImageJ software. The wound closure was calculated as the percentage of the healed epithelial area at each time point/initial scratch area. Each experiment was done in replicates with at least three biological replicates.

### Nanopore RNA sequencing and genome-wide cDNA microarray analysis

The normal corneal epithelium and regenerative corneal epithelium (16 h after a large wound) were collected for the third generation Nanopore-sequencing. 1 μg total RNA was prepared for cDNA libraries using protocol provided by Oxford Nanopore Technologies (ONT). Briefly, SuperScript IV First-Strand Synthesis System (Invitrogen) was used for full length mRNA reverse transcription and following cDNA PCR for 14 circles with LongAmp Tag (NEB). The PCR products were then subjected to FFPE DNA repair and end-repair (NEB) steps and following adaptor ligation using T4 DNA ligase (NEB). Agencourt XP beads were used for DNA purification according to the ONT protocol. The final cDNA libraries were added to FLO-MIN109 flowcells and were run on PromethION platform at Beijing Biomarker Technologies (Beijing, China). Full length reads were mapped to the reference transcriptome sequence. Reads with match quality above 5 were further used to quantify. Expression levels were estimated by reads per gene/transcript per 10,000 reads mapped. The resulting *p* values were adjusted using the Benjamini and Hochberg’s approach for controlling the false discovery rate. Transcripts with a FDR < 0.01 and fold change ≥2 found by DESeq were assigned as differentially expressed. KOBAS software was used to test the statistical enrichment of differential expression transcripts in KEGG pathways.

These published genome-wide cDNA microarray raw data that compare regenerative corneal epithelium (42 h after a large wound) with normal corneal epithelium of rat are available and permitted to use^19^. Genes relating to this study are selected and listed in Supplementary Tables 1–3. Differentially expressed genes were identified by comparing regenerative with normal corneal epithelium, and only genes with log2 fold change >1 or < −1 and −log10 *p*-value >2 were considered significant. According to these differentially expressed genes of interest, volcano graphs were plotted using GraphPad Prism software (Version 6.0). The black points represent genes with statistical significance, while gray points represent genes without statistical significance. These key genes related to the present study were emphasized by different color dots. All these YAP target genes involved in the present study were cited from the previous reports^29,45,46^.

### Quantification and statistical analysis

Quantifications were performed from at least three independent experiments, biological replicates or sections. Data were analyzed and statistics performed. Unpaired or paired two-tailed Student’s *t*-test and one-way ANOVA with Dunnett’s test were used for pairwise comparisons and multi-group comparison, respectively. Mean ± SD were represented, and significance was set as *p < 0.05, **p < 0.01, and ***p < 0.001, unless otherwise indicated. All analysis was performed with GraphPad Prism software (Version 6.0).

### Reporting summary

Further information on experimental design is available in the Nature Research Reporting Summary linked to this paper.

## Supplementary information

Supplementary Information

Reporting Summary Checklist

## Data Availability

The RNA-seq data were deposited in NCBI Sequence Read Archive (SRA) under the BioProject accession number PRJNA 669218. All data supporting the conclusions of this study are either provided in this published paper (and its Supplementary Information files) or available from the authors upon reasonable request.

## References

[1] Yanger K (2014). Adult hepatocytes are generated by self-duplication rather than stem cell differentiation. Cell Stem Cell.

[2] Chen F (2020). Broad distribution of hepatocyte proliferation in liver homeostasis and regeneration. Cell Stem Cell.

[3] Jadhav U (2017). Dynamic reorganization of chromatin accessibility signatures during dedifferentiation of secretory precursors into Lgr5+ intestinal stem cells. Cell Stem Cell.

[4] Tetteh PW (2016). Replacement of lost Lgr5-positive stem cells through plasticity of their enterocyte-lineage daughters. Cell Stem Cell.

[5] Tata PR (2013). Dedifferentiation of committed epithelial cells into stem cells in vivo. Nature.

[6] Wells JM, Watt FM (2018). Diverse mechanisms for endogenous regeneration and repair in mammalian organs. Nature.

[7] Merrell AJ, Stanger BZ (2016). Adult cell plasticity in vivo: de-differentiation and transdifferentiation are back in style. Nat. Rev. Mol. Cell Biol..

[8] Yoon JJ, Ismail S, Sherwin T (2014). Limbal stem cells: Central concepts of corneal epithelial homeostasis. World J. Stem Cells.

[9] Di Girolamo N (2015). Moving epithelia: Tracking the fate of mammalian limbal epithelial stem cells. Prog. Retin. Eye Res..

[10] Vauclair S (2007). Corneal epithelial cell fate is maintained during repair by Notch1 signaling via the regulation of vitamin A metabolism. Dev. Cell.

[11] Chang CY (2008). Acute wound healing in the human central corneal epithelium appears to be independent of limbal stem cell influence. Invest. Ophthalmol. Vis. Sci..

[12] West JD, Dorà NJ, Collinson JM (2015). Evaluating alternative stem cell hypotheses for adult corneal epithelial maintenance. World J. Stem Cells.

[13] Zheng Y, Pan D (2019). The Hippo signaling pathway in development and disease. Dev. Cell..

[14] Totaro A, Panciera T, Piccolo S (2018). YAP/TAZ upstream signals and downstream responses. Nat. Cell Biol..

[15] Moya IM, Halder G (2019). Hippo-YAP/TAZ signalling in organ regeneration and regenerative medicine. Nat. Rev. Mol. Cell Biol..

[16] Elbediwy A (2016). YAP and TAZ in epithelial stem cells: a sensor for cell polarity, mechanical forces and tissue damage. Bioessays.

[17] Raghunathan VK (2014). Involvement of YAP, TAZ and HSP90 in contact guidance and intercellular junction formation in corneal epithelial cells. PLoS ONE.

[18] Kasetti RB (2016). Study of corneal epithelial progenitor origin and the Yap1 requirement using keratin 12 lineage tracing transgenic mice. Sci. Rep..

[19] Bettahi I (2014). Genome-wide transcriptional analysis of differentially expressed genes in diabetic, healing corneal epithelial cells: hyperglycemia-suppressed TGFβ3 expression contributes to the delay of epithelial wound healing in diabetic corneas. Diabetes.

[20] Kim M (2015). Transcriptional co-repressor function of the hippo pathway transducers YAP and TAZ. Cell Rep..

[21] Goto H (2018). Loss of Mob1a/b in mice results in chondrodysplasia due to YAP1/TAZ-TEAD-dependent repression of SOX9. Development.

[22] Liu-Chittenden Y (2012). Genetic and pharmacological disruption of the TEAD-YAP complex suppresses the oncogenic activity of YAP. Genes Dev..

[23] Park M (2019). Visualizing the contribution of keratin-14+ limbal epithelial precursors in corneal wound healing. Stem Cell Rep..

[24] Playford MP (2008). Focal adhesion kinase regulates cell-cell contact formation in epithelial cells via modulation of Rho. Exp. Cell Res..

[25] Ma Y (2013). Focal adhesion kinase regulates intestinal epithelial barrier function via redistribution of tight junction. Biochim. Biophys. Acta.

[26] Kanellos G, Frame MC (2016). Cellular functions of the ADF/cofilin family at a glance. J. Cell Sci..

[27] Yin J, Lu J, Yu F-SX (2008). Role of small GTPase Rho in regulating corneal epithelial wound healing. Invest. Ophthalmol. Vis. Sci..

[28] Yin J, Yu F-SX (2008). Rho kinases regulate corneal epithelial wound healing. Am. J. Physiol. Cell Physiol..

[29] Nardone G (2017). YAP regulates cell mechanics by controlling focal adhesion assembly. Nat. Commun..

[30] Baddour JA, Sousounis K, Tsonis PA (2012). Organ repair and regeneration: an overview. Birth Defects Res. C. Embryo Today.

[31] Majo F (2008). Oligopotent stemcells are distributed throughout the mammalian ocular surface. Nature.

[32] Dorà NJ (2015). Lineage tracing in the adult mouse corneal epithelium supports the limbal epithelial stem cell hypothesis with intermittent periods of stem cell quiescence. Stem Cell Res..

[33] Richardson A (2017). Keratin-14-positive precursor cells spawn a population of migratory corneal epithelia that maintain tissue mass throughout life. Stem Cell Rep..

[34] Park M (2019). Peripheral (not central) corneal epithelia contribute to the closure of an annular debridement injury. Proc. Natl. Acad. Sci. USA.

[35] Nasser W (2018). Corneal-committed cells restore the stem cell pool and tissue boundary following injury. Cell Rep..

[36] Gouveia RM (2019). Assessment of corneal substrate biomechanics and its effect on epithelial stem cell maintenance and differentiation. Nat. Commun..

[37] Nowell CS (2016). Chronic inflammation imposes aberrant cell fate in regenerating epithelia through mechanotransduction. Nat. Cell Biol..

[38] King RS, Newmark PA (2012). The cell biology of regeneration. J. Cell Biol..

[39] Gumbiner BM, Kim NG (2014). The Hippo-YAP signaling pathway and contact inhibition of growth. J. Cell Sci..

[40] Garcia, M. A., Nelson, W. J. & Chavez, N. Cell-cell junctions organize structural and signaling networks. *Cold Spring Harb. Perspect. Biol*. **10**, pii: a029181 (2018). 10.1101/cshperspect.a029181 PMC5773398 28600395

[41] Byrne KM (2016). Bistability in the Rac1, PAK, and RhoA signaling network drives actin cytoskeleton dynamics and cell motility switches. Cell Syst..

[42] Zihni C, Terry SJ (2015). RhoGTPase signalling at epithelial tight junctions: bridging the GAP between polarity and cancer. Int. J. Biochem. Cell Biol..

[43] Ohgushi M, Minaguchi M, Sasai Y (2015). Rho-signaling-directed YAP/TAZ activity underlies the long-term survival and expansion of human embryonic stem cells. Cell Stem Cell.

[44] Li Y (2017). Poly(ethylene glycol)-modified silk fibroin membrane as a carrier for limbal epithelial stem cell transplantation in a rabbit LSCD model. Stem Cell Res. Ther..

[45] Zanconato F (2015). Genome-wide association between YAP/TAZ/TEAD and AP-1 at enhancers drives oncogenic growth. Nat. Cell Biol..

[46] Pattschull G (2019). The Myb-MuvB complex is required for YAP-dependent transcription of mitotic genes. Cell Rep..

