# Supplementary material for: The common YAP activation mediates corneal epithelial regeneration and repair with different-sized wounds
Source: NPJ Regen Med. 2021 Mar 26;6:16. doi: 10.1038/s41536-021-00126-2 (PMC7997881; doi:10.1038/s41536-021-00126-2)
Supplement: Supplementary file 1 — Supplementary Information [file 41536_2021_126_MOESM1_ESM.pdf]

Supplementary Information

The common YAP activation mediates corneal epithelial regeneration and repair with different-sized wounds

Authors

Yijian Li[1,2]†, Lingling Ge[1,2], Xia Chen[1,2,3], Yumei Mao[4], Xianliang Gu[1,2], Bangqi Ren[1,2], Yuxiao Zeng[1,2], Min Chen[1,2], Siyu Chen[1,2], Jinhua Liu[1,2], Yuli Yang[1,2]† and Haiwei Xu[1,2]†

Affiliations

1. Southwest Hospital/Southwest Eye Hospital, Third Military Medical University (Army Medical University), Chongqing 400038, China.

2. Key Lab of Visual Damage and Regeneration & Restoration of Chongqing, Chongqing 400038, China.

3. Southwest University, Chongqing 400038, China.

4. North Sichuan Medical College, Sichuan 637000, China.

† for corresponding authors

Yijian Li E-mail: liyijian2226@163.com

Yuli Yang E-mail: yylxnyy_@163.com

Haiwei Xu E-mail: haiweixu2001@163.com

This Supplementary Information file includes:

Supplementary Fig. 1. Activation of YAP during corneal epithelial regeneration after large wound.

Supplementary Fig. 2. YAP activation mediates LESCs-dependent regeneration through proliferation and migration.

Supplementary Fig. 3. Expressions of LESCs markers during LESCs-dependent regeneration.

Supplementary Fig. 4. YAP-mediated reprogramming of repairing epithelial cells contributes to LESCs-independent repair after small wound.

Supplementary Fig. 5. Loosening of adhesions and disrupted cortical F-actin cytoskeleton during regeneration and repair.

Supplementary Fig. 6. Manipulating ROCK/LIMK/Cofilin pathway regulates YAP activity.

Supplementary Fig. 7. YAP as a key regulator of the assembly of cell junction and cortical F-actin cytoskeleton.

Supplementary Fig. 8. Uncropped images of Western blots.

Supplementary Table 1: Differentially expressed YAP target genes in Genome-wide cDNA microarray

Supplementary Table 2: Differentially expressed YAP regulators in Genome-wide cDNA array

Supplementary Table 3: Differentially expressed genes involving actin dynamics in Genome-wide cDNA array

Supplementary Table 4: Primer sequence

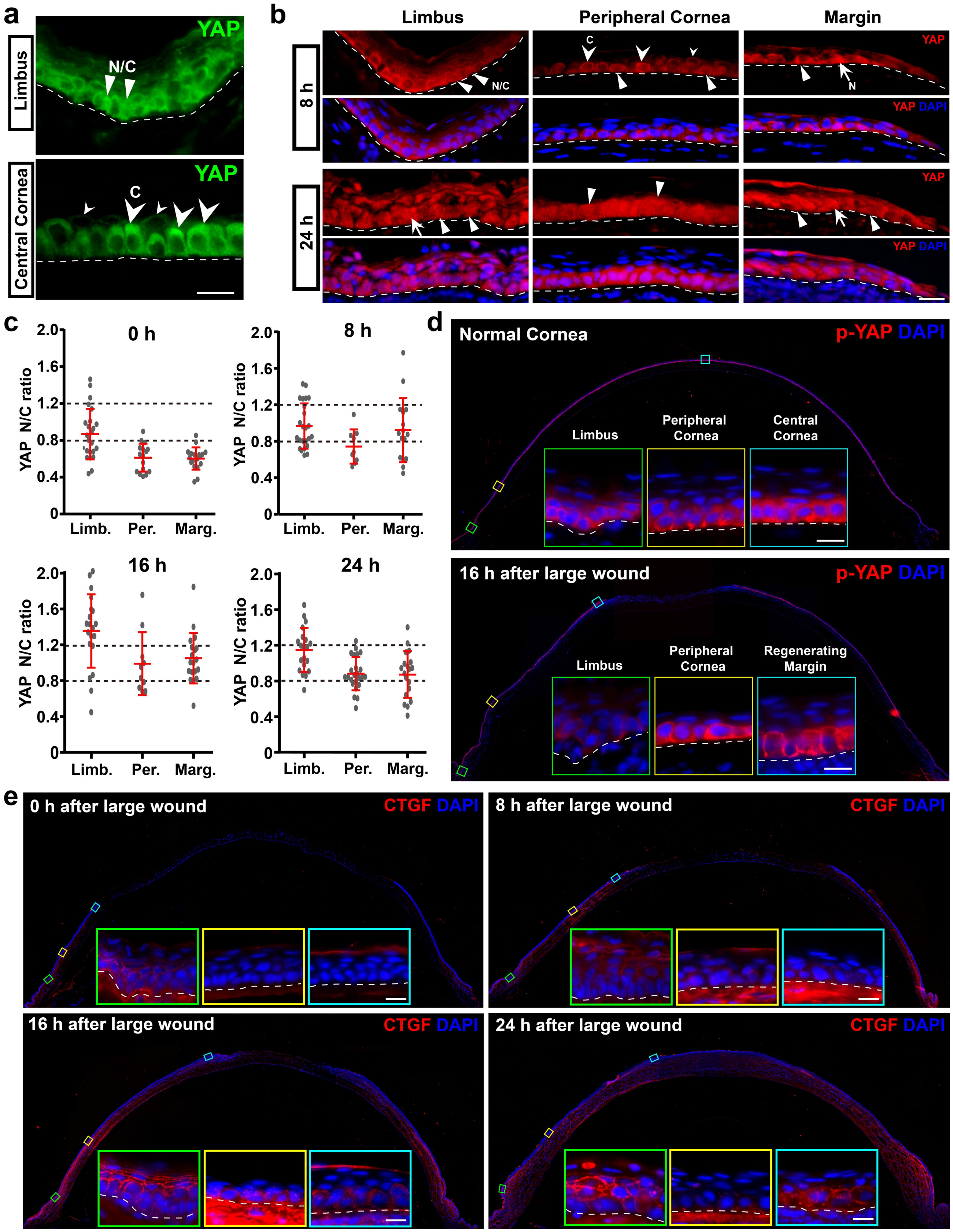

Supplementary Fig. 1. Activation of YAP during corneal epithelial regeneration after large wound. (**a**) YAP localization in limbal and central corneal epithelium of adult rats. (**b**)

Immunofluorescence localization of YAP in limbus, peripheral cornea and regenerative margin at 8 and 24h post wound (pw). Cytoplasmic (C), nuclear (N), and evenly nuclear and cytoplasmic (N/C) YAP distributions are shown. (**c**) The YAP N/C ratio of epithelial cells in limbus, peripheral cornea and regenerative margin at 0, 8, 16, 24h pw. Higher YAP N/C ratio is observed in marginal epithelial cells than peripheral epithelial cells from 8 to 24h pw, especially at 8h pw. (**d**) p-YAP (Ser127) expression during normal homeostasis and regeneration at 16h after large wound. Lower fluorescence intensity of p-YAP staining indicates the activation of YAP in limbal epithelial cells during regeneration. (**e**) The immunofluorescence staining of CTGF, a canonical YAP target gene, during regeneration at 0, 8, 16, 24h pw. Higher fluorescence intensity of CTGF staining is observed in marginal epithelial cells than peripheral epithelial cells during regeneration, especially at 24h pw. The broken white lines identify the epithelial/stromal boundary. Scale bars, 20μm (**a**, **b**, **d**, **e**).

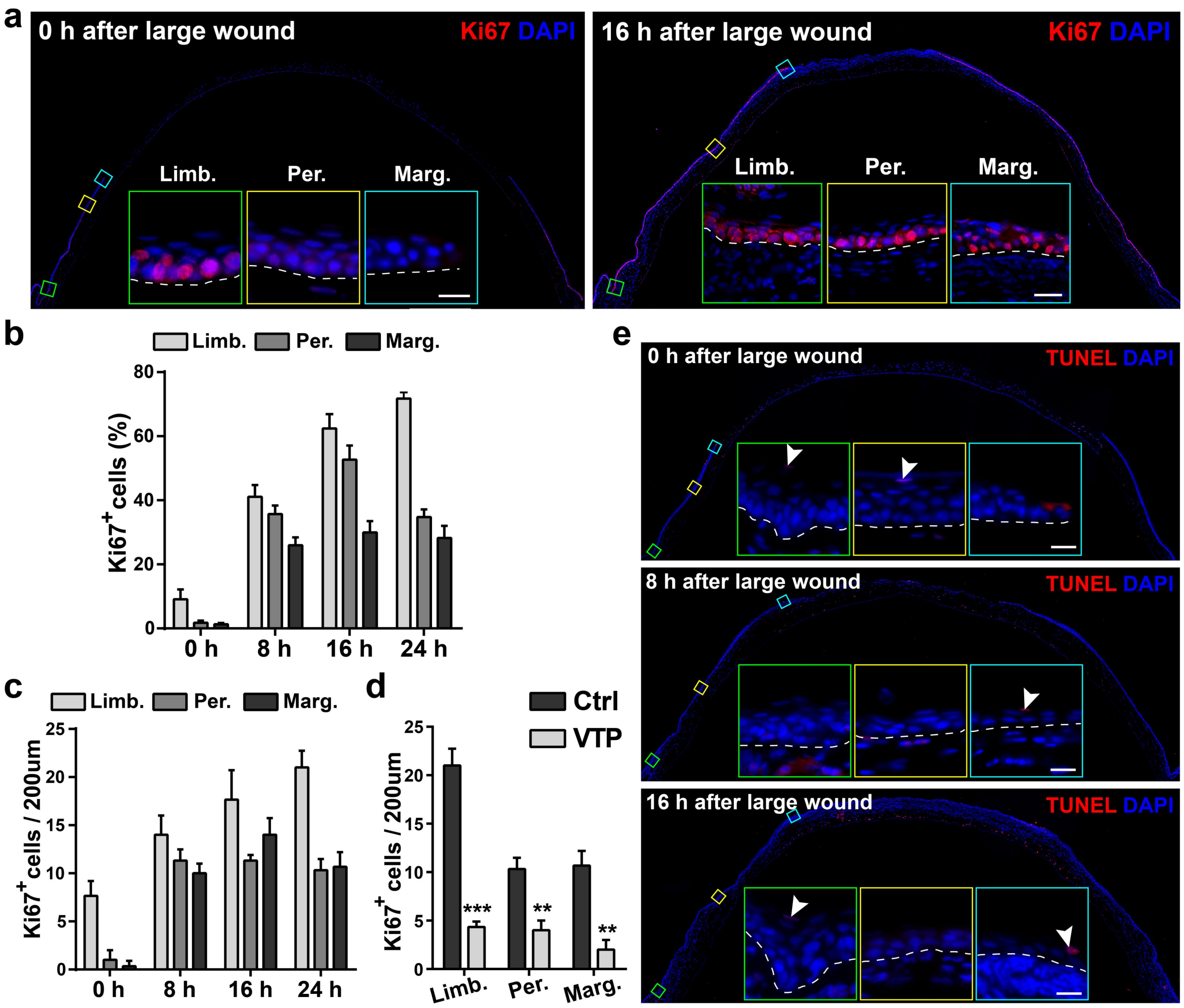

Supplementary Fig. 2. YAP activation mediates LESCs-dependent regeneration through proliferation and migration. (**a**) Proliferation of epithelial cells (Ki67-positive) during regeneration at 0 and 16h pw. (**b**) Percentages of Ki67-positive epithelial cells in limbus, peripheral cornea and regenerative margin during regeneration. (**c**) The numbers of Ki67-positive epithelial cells (per 200μm) in limbus, peripheral cornea and regenerative margin during regeneration. (**d**) The numbers of Ki67-positive epithelial cells (per 200μm) in limbus, peripheral cornea and regenerative margin without or with VTP treatment at 24h pw. (**e**) TUNEL staining of apoptotic cells during regeneration at 0, 8 and 16h pw. There is no difference between normal and regenerative corneal epithelial cells. Arrows point to TUNEL-positive apoptotic cells. Scale bars, 20μm (**a**, **e**). Data are the mean±SD, n=3 sections (**b**, **c**, **d**); $^{**}p < 0.01$, $^{***}p < 0.001$; Student's two-tailed unpaired *t*-test (**d**).

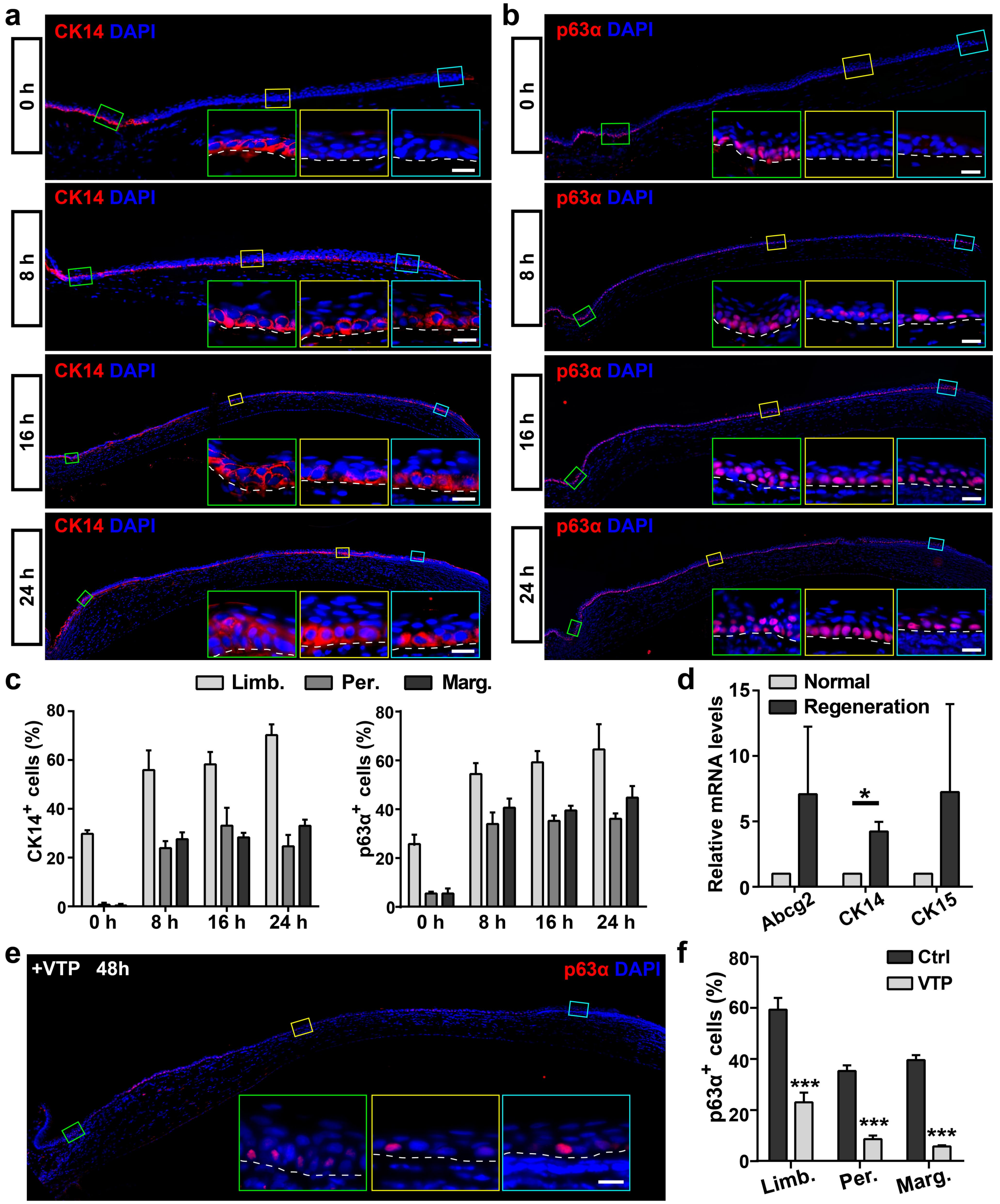

Supplementary Fig. 3. Expressions of LESCs markers during LESCs-dependent regeneration. (**a**, **b**) Immunofluorescence staining of LESCs markers CK14 and p63α during epithelial regeneration at 0, 8, 16 and 24h pw. (**c**) Percentages of CK14 or p63α-positive epithelial cells in limbus, peripheral

cornea and regenerative margin during regeneration. (**d**) RT-qPCR analysis of LESCs marker genes, Abcg2, CK14 and CK15, in normal corneal epithelium and regenerative epithelial cells at 16h pw. (**e**) Immunofluorescence staining of LESCs marker p63α during epithelial regeneration at 48h pw under VTP treatment. (**f**) Percentages of p63α-positive epithelial cells in limbus, peripheral cornea and regenerative margin without or with VTP treatment at 48h pw. Scale bars, 20μm (**a**, **b**, **e**). Data are the mean±SD, n=3 sections (**b**), n=4 sections (**f**), n=3 biological replicates (**d**); $^{*}p < 0.05$, $^{***}p < 0.001$; Student's two-tailed paired *t*-test (**d**) or unpaired *t*-test (**f**).

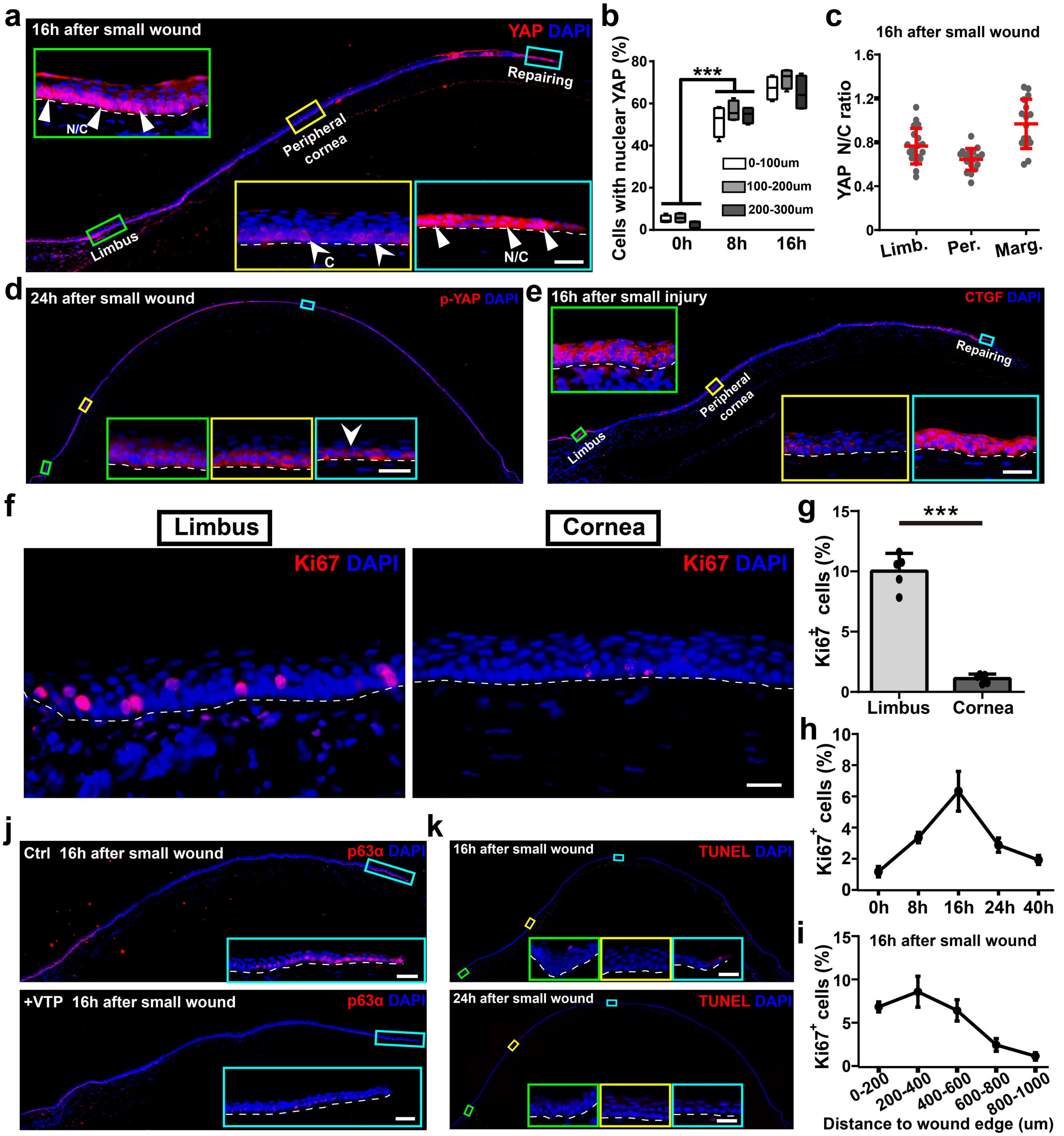

Supplementary Fig. 4. YAP-mediated reprogramming of repairing epithelial cells contributes to LESCs-independent repair after small wound. (**a**) Immunofluorescence localization of YAP in limbus, peripheral cornea and repairing margin at 16h pw. (**b**) Percentages of repairing epithelial cells with N and N/C YAP at different distance to the leading edge of the small wound. (**c**) The YAP N/C ratio of epithelial cells in limbus, peripheral cornea and repairing margin at 16h pw. (**d**, **e**) P-YAP (Ser127) and CTGF stainings during small wound repair. Arrow points to epithelial cells with lower

fluorescence intensity of p-YAP. Higher fluorescence intensity of CTGF staining in repairing epithelial cells indicates YAP activation. (**f**, **g**) Ki67 staining and quantification of epithelial cells in limbus and central cornea during normal homeostasis. (**h**, **i**) Percentage of Ki67-positive repairing epithelial cells at different time points and different distances to the leading edge of the wound. (**j**) Immunofluorescence staining of p63α during epithelial repair at 16h pw in vehicle (Ctrl) and VTP-treated corneas. (**k**) TUNEL staining of apoptotic cells during repair at 16 and 24h pw. Scale bars, 40μm (**a**, **d**, **e**, **f**, **j**, **k**). Data are the mean±SD, n=4 sections (**b**, **c**), n=5 sections (**g**), n=3 sections (**h**, **i**); ***p < 0.001; Student's two-tailed unpaired *t*-test (**b**, **g**).

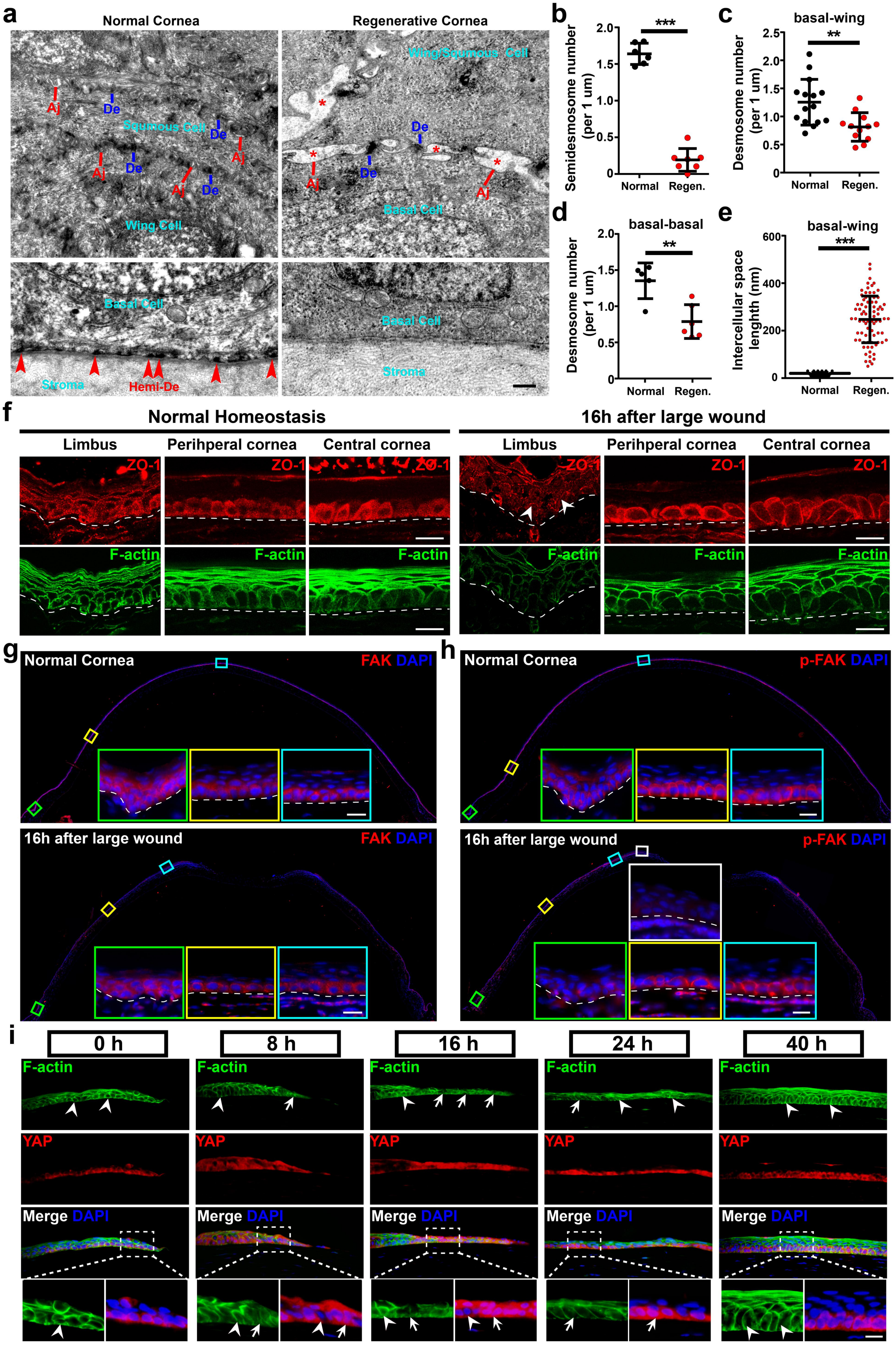
a
Normal Cornea
Regenerative Cornea
Aj
De
Squmous Cell
Wing Cell
Wing/Squmous Cell
Basal Cell
Stroma
Hemi-De
b
Semidesmosome number (per 1 um)
***
Normal
Regen.
c
basal-wing
Desmosome number (per 1 um)
**
d
basal-basal
e
Intercellular space lenghth (nm)
f
Normal Homeostasis
16h after large wound
Limbus
Perihperal cornea
Central cornea
ZO-1
F-actin
g
Normal Cornea
FAK DAPI
16h after large wound
h
p-FAK DAPI
i
0 h
8 h
16 h
24 h
40 h
YAP
Merge DAPI

Supplementary Fig. 5. Loosening of adhesions and disrupted cortical F-actin cytoskeleton during regeneration and repair. (**a**) TEM images of corneal epithelial cells during normal homeostasis and at 16h after large wound (regenerative). Adherens junctions (Aj), desmosome (De) and hemidesmosome (Hemi-De) are shown. Clear intercellular loosening of Aj is indicated with asterisks. (**b-d**) Quantification of Hemi-De and De numbers of corneal epithelial basal cells during normal homeostasis and at 16h after large wound (regen.). (**e**) Quantifications of intercellular space lengths between basal cells and wing cells during normal homeostasis and at 16h after large wound (regen.). (**f**) ZO-1 and fluorescein-phalloidin stainings of limbal and corneal epithelial cells during normal homeostasis and at 16h after large wound (regeneration). (**g**, **h**) FAK and p-FAK (Tyr397, regulator of focal adhesions) stainings of corneal epithelium during normal homeostasis and at 16h after large wound (regeneration). (**i**) YAP and fluorescein-phalloidin stainings of local repairing epithelial cells during corneal epithelial repair after small wound. Arrows and arrowheads indicate present or disrupted cortical actin cytoskeleton, respectively, at the interface between two epithelial cells. Note these repairing epithelial cells with predominantly cytoplasmic YAP and stable cortical F-actin cytoskeleton, which implies that cortical F-actin cytoskeleton suppresses YAP nuclear localization and its activity, or vice versa. Scale bars, 200nm (**a**) or 20μm (**f**, **g**, **h**, **i**). Data are the mean±SD from 3 sections (**b-e**); **p<0.01, ***p<0.001; Student's two-tailed unpaired *t*-test.

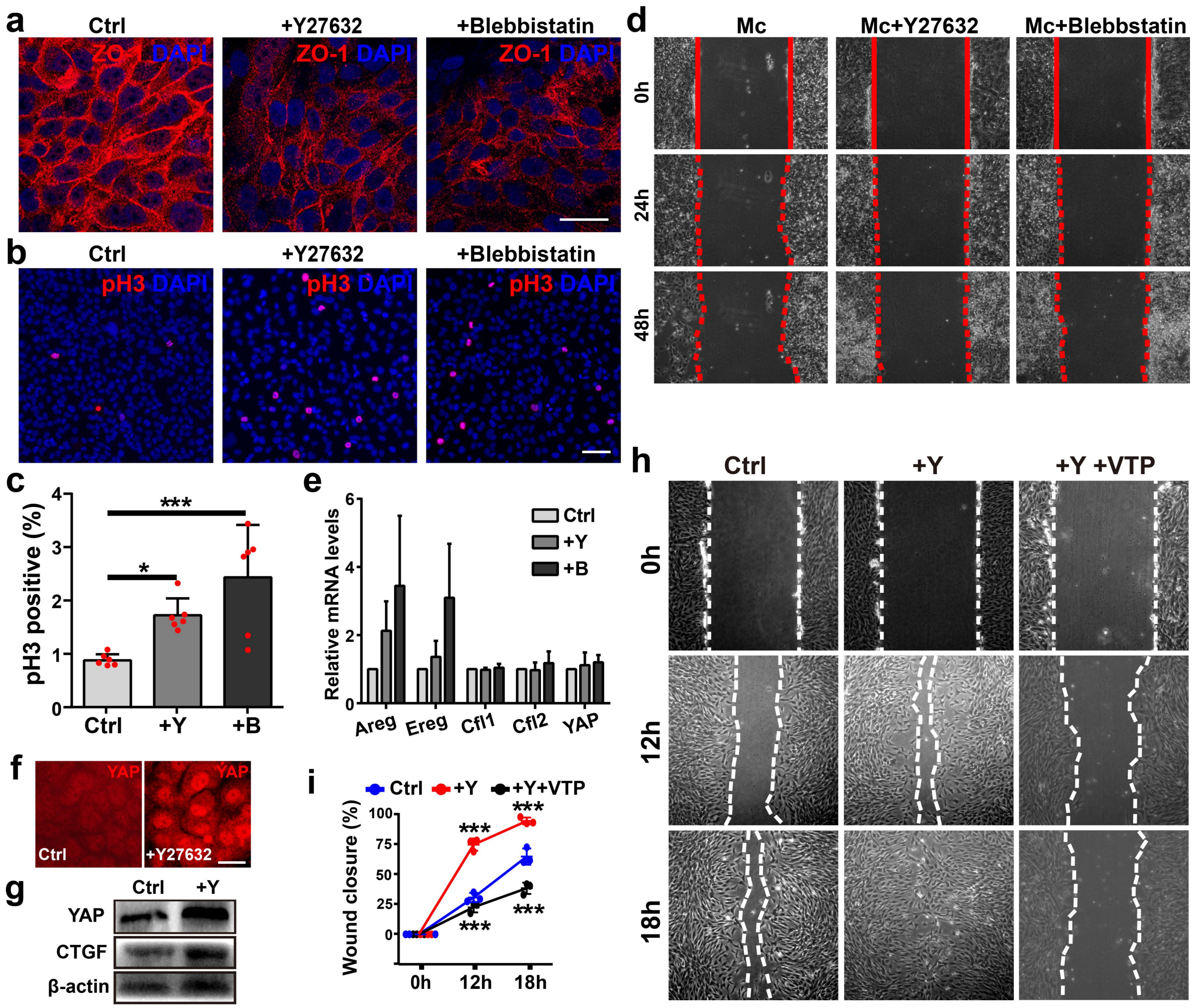

Supplementary Fig. 6. Manipulating ROCK/LIMK/Cofilin pathway regulates YAP activity. (**a**) ZO-1 staining of hCECs under indicated treatments for 8h. (**b, c**) pH3 (a marker of cell proliferation) staining and quantification of hCECs under indicated treatments for 24h. (**d**) hCECs cells are scratch wounded at confluence, and images are captured at 0 and 24h post-scratching under indicated treatments. (**e**) RT-qPCR analysis of genes from hCECs under indicated treatments for 24h. (**f**) YAP staining of hCECs under indicated treatments for 24h. (**g**) Western blotting analysis of YAP and CTGF protein levels from hCECs under indicated treatments for 24h. (**h**) hCECs are scratch wounded at confluence, and images are captured at 0, 12 and 18h post-scratching. (**i**) Quantifications of wound closure at 0, 12 and 18h post-scratching under indicated treatments. +Y vs Ctrl, +Y+VTP vs +Y. Scale bars, 20μm (**a**, **f**) or 60μm (**b**). Data are the mean±SD, n=6 fields from 2 experiments (**c**), n=3

biological replicates (**e**, **i**); *$p<0.05$, ***$p < 0.001$; One-way ANOVA with Dunnett's post test (**c**) or Student's two-tailed unpaired *t*-test (**i**). Ctrl, 0.1% DMSO; Y27632, 20μM (**a**) or 10μM (**b**-**i**); Blebbistatin, 30μM (**a**) or 20μM (**b**-**i**); Mc, mitomycin C, 5μg/mL (**d**); VTP, verteporfin, 5μM (**e**, **h**, **i**).

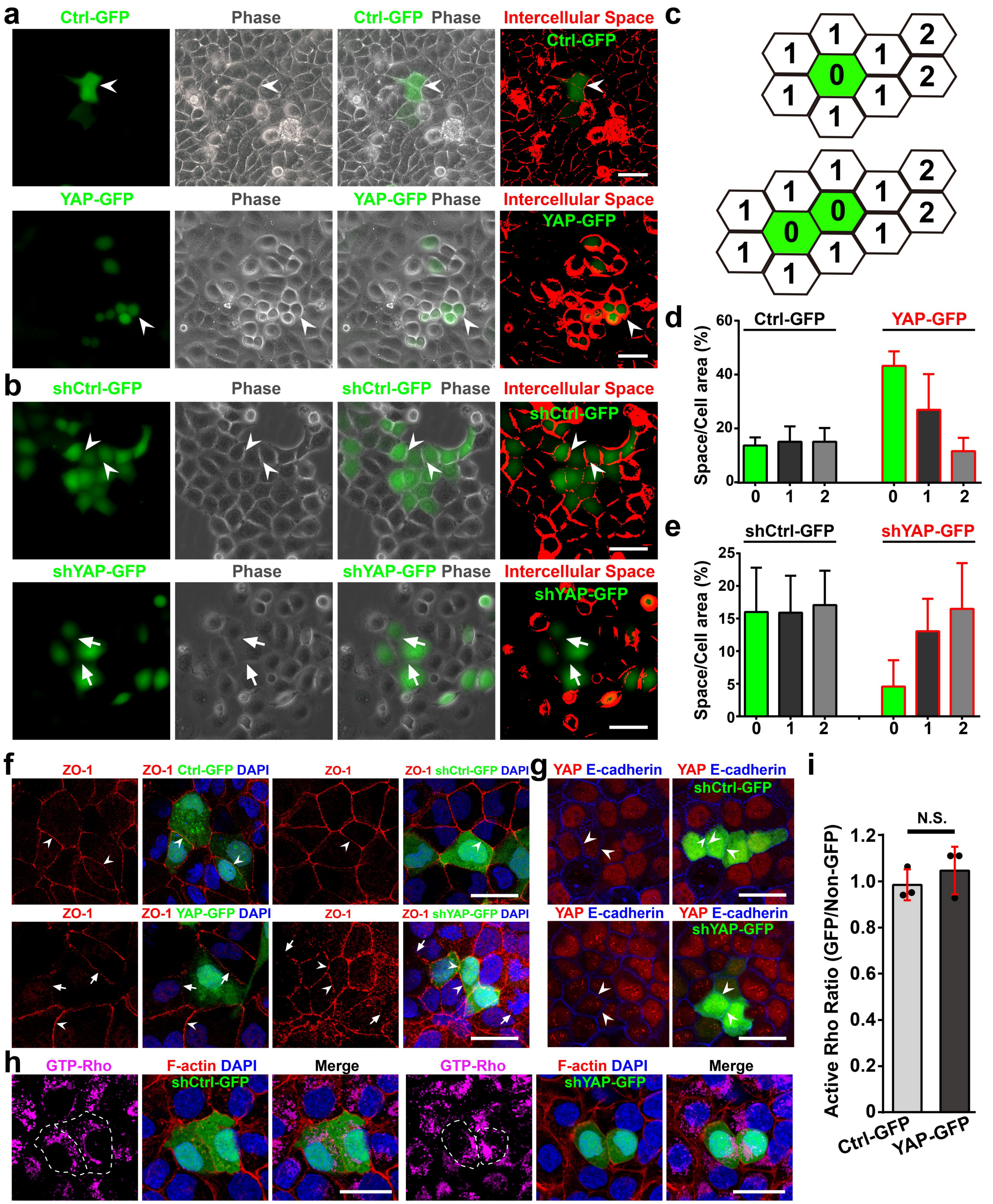

Supplementary Fig. 7. YAP as a key regulator of the assembly of cell junction and cortical F-actin cytoskeleton. (**a**) YAP-overexpressing hCECs (YAP-GFP) and hCECs infected with control lentivirus (Ctrl-GFP) are co-cultured with uninfected hCECs (without GFP). (**b**) YAP-knockdown

hCECs (shYAP-GFP) and hCECs infected with control lentivirus (shCtrl-GFP) are co-cultured with uninfected hCECs (without GFP). Intercellular spaces are shown. Arrows and arrowheads indicate intercellular loosening and tightening, respectively. (**c**) Graphical representation of lentivirus infected hCECs (with GFP) and the neighboring uninfected hCECs (without GFP). The lentivirus infected hCECs (green) are defined as the cell 0, and its neighboring uninfected hCECs are defined as the cell 1 or 2 (white) as shown. (**d**) Quantification of intercellular space area/total cellular area of the cell 0, 1 and 2 in YAP-overexpressing hCECs (YAP-GFP) and hCECs infected with control lentivirus (Ctrl-GFP) that co-cultured with uninfected hCECs. (**e**) Quantification of intercellular space area/total cellular area of the cell 0, 1 and 2 in YAP-knockdown hCECs (shYAP-GFP) and hCECs infected with control lentivirus (shCtrl-GFP) that co-cultured with uninfected hCECs. (**f**) Immunostaining of hCECs for ZO-1. Arrows and arrowheads indicate present or disrupted ZO-1 staining, respectively, at the interface between two hCECs. (**g**) Immunostaining of hCECs for YAP and E-cadherin. Arrows indicate present E-cadherin staining at the interface between two infected hCECs. (**h**) Staining of hCECs for active GTP-bound Rho and cortical actin cytoskeleton. The broken white lines show GFP-positive infected hCECs. (**i**) The GTP-bound active Rho ratio of immunofluorescence intensity between GFP-positive infected hCECs (shCtrl or shYAP) and GFP-negative uninfected hCECs. Scale bars, 20µm (**a**, **b**, **f**, **g**, **h**). Data are the mean±SD from at least 3 fields (**d**, **e**, **i**); Student's two-tailed unpaired *t*-test (**i**).

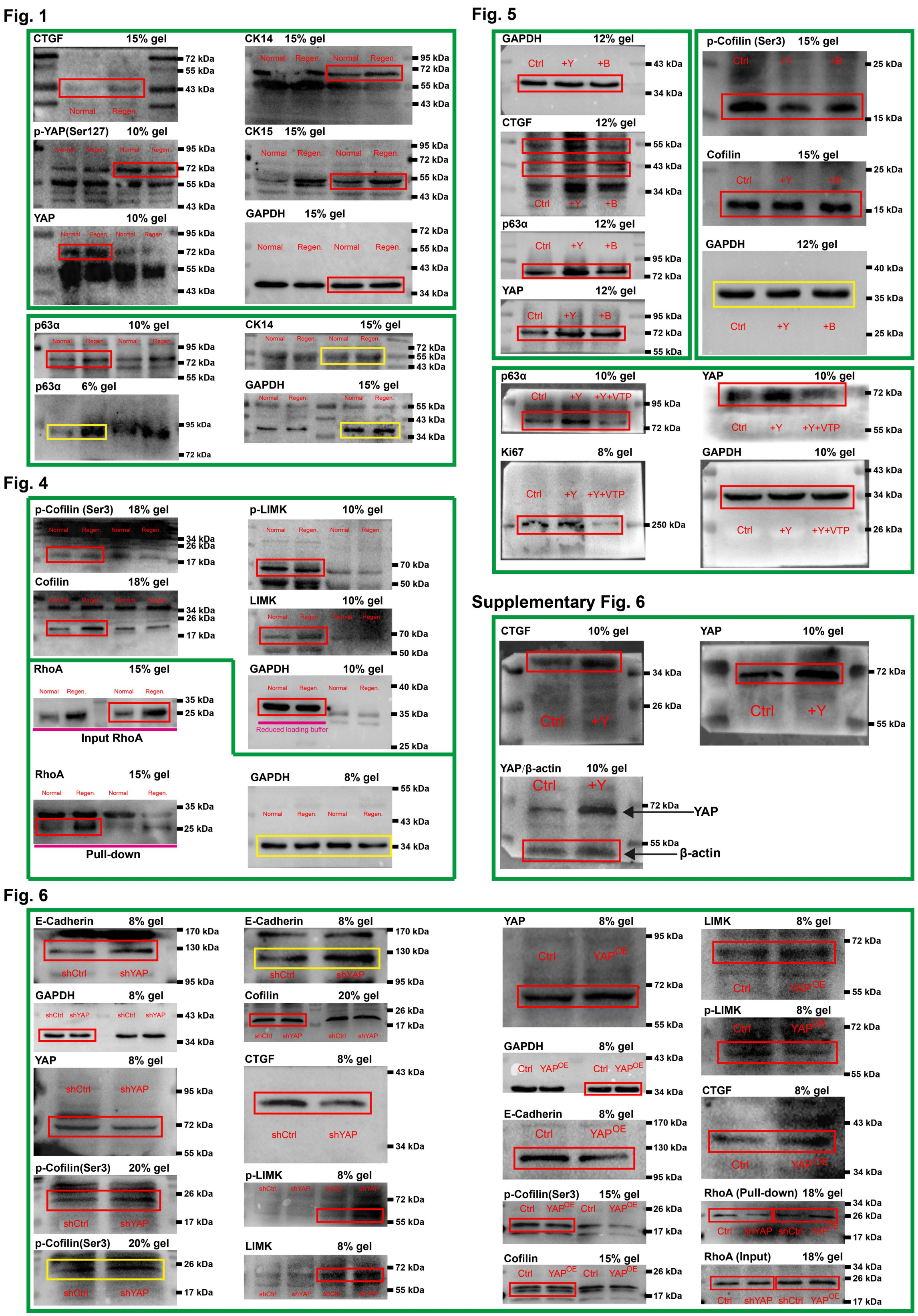

Supplementary Fig. 8. Uncropped images of Western blots. Red boxes indicate areas that are cropped. Yellow boxes show some biological replicates that not displayed in the main and supplementary figures.

Supplementary Table 1: Differentially expressed YAP target genes in rat corneal epithelia detected by Genome-wide cDNA array

Gene Symbol	Gene Title	p-value	Fold-Change
Adrb2	adrenergic, beta-2-, receptor, surface	0.00147352	-2.02606
Aimp2	aminoacyl tRNA synthetase complex-interacting multifunctional protein 2	3.48E-06	1.98309
Akap12	A kinase (PRKA) anchor protein 12	0.000482663	3.29052
Anxa3	annexin A3	0.0196051	1.84247
Areg	amphiregulin	0.00110983	7.45906
ARHGAP29	Rho GTPase activating protein 29	0.0151884	2.2045
Asap1	ArfGAP with SH3 domain, ankyrin repeat and PH domain 1	0.729628	-1.05582
Basp1 /// LOC100910172	brain abundant, membrane attached signal protein 1 /// brain acid soluble protei	0.000631711	5.47924
Bcat1	branched chain aminotransferase 1, cytosolic	3.75E-08	38.193
Birc5	baculoviral IAP repeat-containing 5	2.73E-05	2.32205
Bub1b	budding uninhibited by benzimidazoles 1 homolog, beta (S. cerevisiae)	0.00281907	1.8176
C1qbp	complement component 1, q subcomponent binding protein	2.78E-06	2.07061
Casc5	cancer susceptibility candidate 5	0.00441116	1.74406
Cdca4	cell division cycle associated 4	1.82E-06	1.72699
Cdca5	cell division cycle associated 5	0.0271652	1.32247
Cdca8	cell division cycle associated 8	7.75E-05	2.03204
Cd44	Cd44 molecule	1.30E-08	5.09187
Ccna2	cyclin A2	8.61E-06	2.49512
Ccne1	cyclin E1	1.65E-05	2.71843
Cenpf	centromere protein F	0.00742973	1.45924
Cenpv	centromere protein V	1.48E-06	6.69513
Ckap4	cytoskeleton-associated protein 4	0.197376	1.12398
Col12a1	collagen, type XII, alpha 1	0.00319792	5.82385
Crim1	cysteine rich transmembrane BMP regulator 1 (chordin like)	2.26E-05	-2.08087
Ctgf	connective tissue growth factor	0.890199	1.11045
CTNND2	catenin (cadherin-associated protein), delta 2 (neural plakophilin-related arm-repeat protein)	1.47E-05	-4.41052
Cxxc5	CXXC finger 5	1.31E-06	-2.21035
Cyp20a1	cytochrome P450, family 20, subfamily a, polypeptide 1	5.84E-07	2.83731
Cyr61	cysteine-rich, angiogenic inducer, 61	0.386552	1.38006
Dab2	disabled homolog 2 (Drosophila)	0.000108077	3.50393
Dars2	aspartyl-tRNA synthetase 2 (mitochondrial)	1.57E-06	-2.82348
Ddah1	dimethylarginine dimethylaminohydrolase 1	0.00147614	2.83918
Ddit4	DNA-damage-inducible transcript 4	0.0143356	2.64123
Ddx21	DEAD (Asp-Glu-Ala-Asp) box polypeptide 21	7.52E-05	1.72899
Ddx56	DEAD (Asp-Glu-Ala-Asp) box polypeptide 56	5.79E-06	2.03632
Diaph3	diaphanous homolog 3 (Drosophila)	0.00421825	1.58705
Dusp14	dual specificity phosphatase 14	0.00227239	2.50226
Dut	Deoxyuridine triphosphatase	0.783568	1.05417
Eftud2	elongation factor Tu GTP binding domain containing 2	0.00330574	1.52294
Edn1	endothelin 1	0.898924	1.04323

Eif5a2	eukaryotic translation initiation factor 5A2	7.38E-06	2.7187
Ercc6l	excision repair cross-complementing rodent repair deficiency complementation group 6 -	0.00247436	1.77454
Ereg	epiregulin	0.0383121	2.05837
Ets1	v-ets erythroblastosis virus E26 oncogene homolog 1 (avian)	2.29E-08	25.5484
Etv5	ets variant 5	0.000356225	-2.78746
Fam89a	family with sequence similarity 89, member A	0.0574377	-1.7348
Fjx1	four jointed box 1 (Drosophila)	3.36E-05	-4.87391
Flna	filamin A, alpha	2.64E-06	2.69381
Fosl1	fos-like antigen 1	4.08E-06	20.8162
FSTL1	follistatin-like 1	6.52E-10	52.3175
Gadd45a	growth arrest and DNA-damage-inducible, alpha	0.740965	1.11077
Ggh	gamma-glutamyl hydrolase (conjugase, folylpolygammaglutamyl hydrolase)	0.0014412	1.78904
Gja1	gap junction protein, alpha 1	0.0185606	1.48876
Gnl3	guanine nucleotide binding protein-like 3 (nucleolar)	8.84E-06	1.93726
Gpatch4	G patch domain containing 4	0.00042238	1.79463
Hbegf	heparin-binding EGF-like growth factor	0.374927	1.19703
Igfbp3	insulin-like growth factor binding protein 3	0.000128364	23.5302
Il12a	interleukin 12a	2.19E-06	-2.80154
Iqgap3	IQ motif containing GTPase activating protein 3	0.000177897	2.27953
Itgav	integrin, alpha V	8.38E-05	3.38168
Itgb2	integrin, beta 2	0.000659454	2.21711
Itgb3	Integrin, beta 3	0.0109706	1.9015
Kif2c	kinesin family member 2C	0.0014215	1.6793
Kif18b /// LOC100363850	kinesin family member 18B /// kinesin-like protein KIF18B-like	0.00738729	1.62731
Kif20b	kinesin family member 20B	0.0029617	1.66742
Klf9	Kruppel-like factor 9	5.39E-05	-2.1726
Krt15	keratin 15	8.37E-07	19.0848
Lmnb2	Lamin B2	0.296764	1.09875
Lrig3	leucine-rich repeats and immunoglobulin-like domains 3	0.000151805	-2.24311
Lrrc8c	leucine rich repeat containing 8 family, member C	6.50E-06	13.5618
Map3k1	mitogen activated protein kinase kinase kinase 1	1.32E-05	-2.00927
Matn2	matrilin 2	8.24E-06	-3.20597
Mcm3	minichromosome maintenance complex component 3	0.00204621	1.71428
Mcm7	minichromosome maintenance complex component 7	0.000836375	1.95659
Mre11a	MRE11 meiotic recombination 11 homolog A (S. cerevisiae)	0.00020416	1.5409
Mrpl52	mitochondrial ribosomal protein L52	0.0146531	-1.55955
Mrrf	mitochondrial ribosome recycling factor	4.52E-05	-1.79077
Mthfd1l	methylenetetrahydrofolate dehydrogenase (NADP+ dependent) 1-like	0.000157111	2.59075
Myc	myelocytomatosis oncogene	0.000360882	3.93423
NAA25	N(alpha)-acetyltransferase 25, NatB auxiliary subunit	4.79E-05	1.92514
Ncl	nucleolin	3.05E-06	1.91051
Ndrg1	N-myc downstream regulated gene 1	1.56E-05	2.71964
NEDD4L	neural precursor cell expressed, developmentally down-regulated 4-like	5.13E-06	-2.89176
Nfkb1	nuclear factor of kappa light polypeptide gene enhancer in B-cells 1	5.55E-08	2.0878
Nuf2	NUF2, NDC80 kinetochore complex component, homolog (S. cerevisiae)	0.000169711	1.81876

Nup85	nucleoporin 85	0.000325923	1.77091
Nup93	nucleoporin 93	7.19E-06	2.2239
Pabpc4	poly(A) binding protein, cytoplasmic 4	4.66E-09	3.26574
Pak1ip1	PAK1 interacting protein 1	3.08E-05	2.04428
Pank2	pantothenate kinase 2 (Hallervorden-Spatz syndrome)	0.135304	1.24729
Pcdh20	protocadherin 20	0.787515	-1.02722
PHACTR1	phosphatase and actin regulator 1	0.0488209	2.20358
Phf17	PHD finger protein 17	0.000229113	-2.19523
Pkp4	plakophilin 4	7.17E-05	-2.13152
Plau	plasminogen activator, urokinase	0.00013576	8.40578
Pmp22	peripheral myelin protein 22	0.000338384	7.84143
Pprc1	peroxisome proliferator-activated receptor gamma, coactivator-related 1	0.000217579	1.71267
Prmt5	protein arginine methyltransferase 5	0.000290494	1.57311
Ptpla	protein tyrosine phosphatase-like (proline instead of catalytic arginine), member a	3.00E-06	3.69015
Ptrh2	peptidyl-tRNA hydrolase 2	4.76E-05	1.60351
Rab11fip1	RAB11 family interacting protein 1 (class I)	0.000271432	-1.59863
Ralgps2	Ral GEF with PH domain and SH3 binding motif 2	2.79E-06	-2.62323
Rcan1	regulator of calcineurin 1	0.864524	1.02368
Rcc1	regulator of chromosome condensation 1	0.00350561	1.51438
Rnd3	Rho family GTPase 3	0.711764	-1.11062
Rrm2	ribonucleotide reductase M2	5.60E-05	2.30755
Rrs1	RRS1 ribosome biogenesis regulator homolog (S. cerevisiae)	5.85E-05	1.68755
Rtn4ip1	reticulon 4 interacting protein 1	0.464122	-1.08671
Ruvbl2	RuvB-like 2 (E. coli)	2.22E-06	2.09995
Schip1	schwannomin interacting protein 1	1.82E-05	2.91973
Serpine1	serine (or cysteine) peptidase inhibitor, clade E, member 1	0.000614404	7.68737
Sertad4	SERTA domain containing 4	0.000323915	2.36089
Sf3b3	splicing factor 3b, subunit 3	0.0050418	1.35968
Sgcg	sarcoglycan, gamma (dystrophin-associated glycoprotein)	0.000137642	-2.39795
Shcbp1	Shc SH2-domain binding protein 1	6.91E-06	3.65572
Shmt2	serine hydroxymethyltransferase 2 (mitochondrial)	0.000258225	1.8662
Slc1a5	solute carrier family 1 (neutral amino acid transporter), member 5	6.37E-07	8.67215
Slit2	slit homolog 2 (Drosophila)	0.0287065	1.52581
Smc3	structural maintenance of chromosomes 3	0.141063	-1.18389
Soat1	sterol O-acyltransferase 1	2.52E-05	2.6513
Ssr3	signal sequence receptor, gamma	0.945555	-1.00762
Tgfb2	Transforming growth factor, beta 2	0.018473	6.49337
Thg1l	tRNA-histidine guanylyltransferase 1-like (S. cerevisiae)	6.96E-06	2.0519
Timm8a1	translocase of inner mitochondrial membrane 8 homolog a1 (yeast)	3.33E-08	3.59029
Timm10	translocase of inner mitochondrial membrane 10 homolog (yeast)	0.000674357	1.51423
Tk1	thymidine kinase 1, soluble	5.83E-05	1.95723
TLN2	talin 2	8.34E-06	-2.376
Tnfrsf12a	tumor necrosis factor receptor superfamily, member 12a	2.97E-07	12.0719
Tnnt2	troponin T type 2 (cardiac)	0.00104754	2.9669
Tns1	Tensin 1	0.000205201	2.41127

Top2a	topoisomerase (DNA) II alpha	0.000676888	1.56239
Trim14	tripartite motif-containing 14	9.75E-06	-3.58377
Troap	trophinin associated protein	0.00414869	1.5794
Tubb6	tubulin, beta 6	6.04E-07	4.99431
Ube2g2	ubiquitin-conjugating enzyme E2G 2 (UBC7 homolog, yeast)	0.0262231	-1.52036
Uck2	uridine-cytidine kinase 2	0.000305592	2.04776
Vcl	vinculin	9.27E-07	2.42985
Wwc1	WW and C2 domain containing 1	9.10E-06	-2.25835
Wwc2	WW and C2 domain containing 2	0.0210069	1.34082
Zcchc11	zinc finger, CCHC domain containing 11	7.96E-05	-3.23561
Zyx	zyxin	0.000146799	2.73943
Zwilch	Zwilch, kinetochore associated, homolog (Drosophila)	1.52E-05	2.45154

Supplementary Table 2: Differentially expressed YAP regulators in rat corneal epithelia detected by Genome-wide cDNA array

Gene Symbol	Gene Title	p-value	Fold-Change
**Aurka**	aurora kinase A	3.79E-06	3.47476
**Aurkb**	aurora kinase B	1.63E-05	2.01251
**Cav1**	caveolin 1, caveolae protein	1.95E-05	2.76457
**Otud1**	OTU domain containing 1	0.957281	1.00991
**Mybl2**	myeloblastosis oncogene-like 2	5.57E-06	4.12557
**Nup37**	nucleoporin 37	0.000410066	1.65477
**Pak1**	p21 protein (Cdc42/Rac)-activated kinase 1	2.09E-06	4.4115
**Pdlim5**	PDZ and LIM domain 5	0.0376198	1.41414
**Pdlim7**	PDZ and LIM domain 7	7.33E-07	6.94797
**Dlg1**	discs, large homolog 1 (Drosophila)	0.00292217	-1.66528
**Dlg2**	discs, large homolog 2 (Drosophila)	0.00121931	-1.7687
**Eny2**	enhancer of yellow 2 homolog (Drosophila)	1.37E-05	-2.20013
**Fat4**	FAT tumor suppressor homolog 4 (Drosophila)	0.000470835	-6.84018
**Ptpn14**	Protein tyrosine phosphatase, non-receptor type 1	8.86E-05	-3.07341

Note: Gene symbols with red font were putative YAP activators, while gene symbols with dark blue font were putative YAP suppressors.

Supplementary Table 3: Differentially expressed genes involving actin dynamics in rat corneal epithelia detected by Genome-wide cDNA array

Gene Symbol	Gene Title	p-value	Fold-Change
Cfl2	cofilin 2, muscle	0.000294344	2.35606
Cfl1	cofilin 1, non-muscle	8.25E-09	5.19956
Pfn2	profilin 2	0.000220917	2.22423
Tmsbl1	thymosin beta-like protein 1	0.000250967	2.89077
Tmsb10	thymosin, beta 10	1.75E-07	4.4638
Flnb	filamin, beta	1.88E-05	3.33849
Flnb	filamin, beta	9.92E-06	2.83481
Flna	filamin A, alpha	2.64E-06	2.69381
Filip1l	filamin A interacting protein 1-like	3.03E-07	21.6655
Fblim1	filamin binding LIM protein 1	9.24E-08	32.9489
Afap1l2	actin filament associated protein 1-like 2	1.31E-08	16.6741
Actn1	actinin, alpha 1	2.98E-13	70.6093
Tnnt2	troponin T type 2 (cardiac)	0.00104754	2.9669
Tnnt3	troponin T type 3 (skeletal, fast)	0.00684873	-1.6248
Tpm1	tropomyosin 1, alpha	5.68E-06	5.509
Tpm2	tropomyosin 2, beta	1.16E-07	19.4279
Tpm3	Tropomyosin 3, gamma	1.27E-08	3.24737
Myh6	myosin, heavy chain 6, cardiac muscle, alpha	0.0725228	1.54215
Myh10	myosin, heavy chain 10, non-muscle	1.64E-05	2.07994
Myh14	myosin, heavy chain 14	1.24E-07	-3.1708
Myo1b	myosin Ib	7.80E-07	5.92759
Myo1d	myosin ID	0.00330499	1.59876
Myo5a	myosin VA	1.30E-06	3.94136
Myo5c	myosin VC	8.13E-07	-7.49105
Myo6	myosin VI	0.761062	-1.05957
MYO10	myosin X	0.0592201	1.5944
Myl12b	myosin, light chain 12B, regulatory	4.17E-08	-2.19339
Myo18a	myosin XVIIIa	0.785951	-1.03369
Mylip	myosin regulatory light chain interacting protein	1.45E-06	-5.13168
LOC100359980	smooth muscle and non-muscle myosin alkali light chain 6B-like	4.82E-06	-2.24725
Fhod1	formin homology 2 domain containing 1	5.50E-07	2.42848
RGD1560248	Similar to formin-like 2 isoform B	3.45E-06	19.0225
FNBP1L	formin binding protein 1-like	0.00306673	-1.62126
FHOD3	formin homology 2 domain containing 3	1.13E-08	-27.2126
Arpc1b	actin related protein 2/3 complex, subunit 1B	4.57E-09	7.12207
Actr3	ARP3 actin-related protein 3 homolog (yeast)	0.525869	-1.12847
Actr2	ARP2 actin-related protein 2 homolog (yeast)	0.00757645	1.684
Wasf1	WAS protein family, member 1	0.000266025	-3.37177
Wasf2	WAS protein family, member 2	0.0816011	-1.35924
Cyfip2	cytoplasmic FMR1 interacting protein 2	1.96E-08	-6.33995

Prpf40a	PRP40 pre-mRNA processing factor 40 homolog A (S. cerevisiae)	0.891271	1.01172
Snrpa	small nuclear ribonucleoprotein polypeptide A	9.32E-05	1.83148
Brwd1	bromodomain and WD repeat domain containing 1	0.0312788	-1.43099
Cobl	cordon-bleu homolog (mouse)	8.26E-06	-2.32093
**Rho Family**			
PAK1	p21 protein (Cdc42/Rac)-activated kinase 1	2.09E-06	4.4115
Pak2	P21 protein (Cdc42/Rac)-activated kinase 2	0.146414	-1.308
PAK3	p21 protein (Cdc42/Rac)-activated kinase 3	4.05E-07	5.72566
Rac1	Ras-related C3 botulinum toxin substrate 1	0.0341235	1.448
Rac2	ras-related C3 botulinum toxin substrate 2 (rho family, small GTP binding protein Rac2)	0.00132182	2.41458
Racgap1	Rac GTPase-activating protein 1	0.0134284	1.53883
Arhgef2	rho/rac guanine nucleotide exchange factor (GEF) 2	2.82E-06	3.62044
Arhgef37	Rho guanine nucleotide exchange factor (GEF) 37	1.06E-05	-2.65323
Arhgef3	Rho guanine nucleotide exchange factor (GEF) 3	2.51E-08	-2.75529
Arhgef26	Rho guanine nucleotide exchange factor (GEF) 26	3.42E-07	-2.43493
Arhgap9	Rho GTPase activating protein 9	3.66E-05	3.85155
Arhgap1	Rho GTPase activating protein 1	0.00830321	-1.54225
Arhgap24	Rho GTPase activating protein 24	0.00140947	-2.10222
Arhgap22	Rho GTPase activating protein 22	3.62E-05	-2.47954
Arhgap27	Rho GTPase activating protein 27	7.07E-05	-2.47925
Arhgap8	Rho GTPase activating protein 8	1.72E-07	3.38596
Arhgap5	Rho GTPase activating protein 5	0.0463025	-1.28023
Arhgap21	Rho GTPase activating protein 21	0.876155	-1.01756
Arhgap39	Rho GTPase activating protein 39	0.308694	1.16016
Arhgap29	Rho GTPase activating protein 29	0.0151884	2.2045
**Cytoskeletal stability and connection to the membrane**			
Ckap4	cytoskeleton-associated protein 4	0.197376	1.12398
Ctnnd2	catenin (cadherin-associated protein), delta 2 (neural plakophilin-related arm-repeat protein)	1.47E-05	-4.41052
Pcdh20	protocadherin 20	0.787515	-1.02722
Sgcg	sarcoglycan, gamma (dystrophin-associated glycoprotein)	0.000137642	-2.39795
**Cell junction**			
Cldn1	claudin 1	8.63E-08	3.10199
Cldnd1	Claudin domain containing 1	0.00180141	-1.76924
Cldn8	claudin 8	3.26E-05	-3.97185
Cldn10	claudin 10	0.00908674	2.14617
Cdh2	cadherin 2	0.0507854	2.59971
Cdh3	cadherin 3	3.16E-08	3.70907
Cdh10	cadherin 10	0.175249	-1.15852
Cdh11	cadherin 11	0.136597	1.50831
Cdh13	cadherin 13	0.00177255	-1.75414
Ctnnd2	catenin (cadherin-associated protein), delta 2 (neural plakophilin-related arm-repeat protein)	1.47E-05	-4.41052

Dsg2	desmoglein 2	0.000182307	1.99282
Dsg1b	desmoglein 1 beta	5.83E-05	1.66909
**FA (focal adhesion)**			
Vcl	vinculin	9.27E-07	2.42985
Tln2	talin 2	8.34E-06	-2.376
Zyx	zyxin	0.000146799	2.73943
Msn	moesin	1.42E-05	2.12415
Rdx	radixin	0.0294191	-1.80416
Itga1	integrin, alpha 1	0.00957881	3.31966
Itgav	integrin, alpha V	8.38E-05	3.38168
Itga6	integrin, alpha 6	3.09E-06	2.98167
Itga11	integrin, alpha 11	9.56E-05	-1.78136
Itgb2	integrin, beta 2	0.000659454	2.21711
Itgb3	Integrin, beta 3	0.0109706	1.9015
Itgb4	integrin, beta 4	2.44E-05	1.96861
Itgb7	integrin, beta 7	0.46256	1.10025
Sla	Src-like adaptor	7.84E-05	-3.00655

Note: Gene symbols with green font were putative YAP target genes.

Supplementary Table 4 : Primer sequence

Rat			Human	
**Gene**	**Sequence 5'-3'**		**Gene**	**Sequence 5'-3'**
Areg_F	ATGACAGCGACCTATCCAA		Areg-F	GGACCTCAATGACACCTA
Areg_R	GCCTCCTTCTTTCTTCTGC		Areg-R	TTCACTTTCCGTCTTGTT
Ereg_F	ATGGACGGCTACTGCTTG		Ereg-F	CATCTTCTACAGGCAGTCC
Ereg_R	GTTCACACCGCAGACCA		Ereg-R	GCAAACAATAGCCATTCA
Birc5_F	TTCATCCACTGCCCTACC		Cfl2_F	TAAGCGATGACAAAAGACAAA
Birc5_R	TCCCAGCCTTCCAGTTC		Cfl2_R	TACAAAGCATATCGGCAATC
Cxxc5-F	ACGGAGATGCTAAAGCGAGTG		Cfl1_F	TTGATTCCTCTTGGGCTGA
Cxxc5-R	CGCCCAGGTAAGGGAAGT		Cfl1_R	GAAGTTGGCAGCATGGG
Klf9-F	GTTGGACCTGAACAAATACCG		Yap-F	AAAGATGAACAAACGTCCAGCAA
Klf9-R	CCAGAGTGGAGGAGGGAGA		Yap-R	AACTGTGAAAGAGGTCAGCAATACATT
Zcchc11-F	AATGCCACTGTGATAGATG		Gapdh-F	CCATGTTCGTCATGGGTGTGA
Zcchc11-R	ATGTGCTCCTTGGATGTT		Gapdh-R	CATGAGTCCTTCCACGATACCA
Ccne1_F	GTGAAAAGCCAGGATAGCA			
Ccne1_R	GTGGGGATGAAAGAGCAG			
Abcg2_F	ACTGAAGAGGACGGTGGA			
Abcg2_R	ACAGACACTACGCTTTGGC			
Krt14_F (CK14)	CGAGATGGAGCAGCAGAA			
Krt14_R (CK14)	ATTGGGCAGATGAAAGGTG			
Krt15_F (CK15)	GAGGTGGAGTGCCGCTAT			
Krt15_R (CK15)	CTTCCCTGACACCAATAGC			
Cfl2_F	TTCTGCACTTTGGCATACTC			
Cfl2_R	ACATCCTATTCATCCGACTCA			
Cfl1_F	CTGTCTCCCTTTCGTTTCC			
Cfl1_R	TGCCTTCTTGCGTTTCTT			
Gapdh_F	AAGCTGGTCATCAATGGGAAAC			
Gapdh_R	TCACCCCATTTGATGTTAGCG			
