# Supplementary material for: The common YAP activation mediates corneal epithelial regeneration and repair with different-sized wounds
Source: NPJ Regen Med. 2021 Mar 26;6:16. doi: 10.1038/s41536-021-00126-2 (PMC7997881; doi:10.1038/s41536-021-00126-2)
Supplement: Supplementary file 2 — Reporting Summary Checklist [file 41536_2021_126_MOESM2_ESM.pdf]

nature research

Corresponding author(s): Yijian Li; Yuli Yang; Haiwei Xu

Last updated by author(s): Dec 31, 2020

Reporting Summary

Nature Research wishes to improve the reproducibility of the work that we publish. This form provides structure for consistency and transparency in reporting. For further information on Nature Research policies, see our Editorial Policies and the Editorial Policy Checklist.

Statistics

For all statistical analyses, confirm that the following items are present in the figure legend, table legend, main text, or Methods section.

n/a	Confirmed	
☐	☒	The exact sample size (n) for each experimental group/condition, given as a discrete number and unit of measurement
☐	☒	A statement on whether measurements were taken from distinct samples or whether the same sample was measured repeatedly
☐	☒	The statistical test(s) used AND whether they are one- or two-sided<br>*Only common tests should be described solely by name; describe more complex techniques in the Methods section.*
☒	☐	A description of all covariates tested
☐	☒	A description of any assumptions or corrections, such as tests of normality and adjustment for multiple comparisons
☐	☒	A full description of the statistical parameters including central tendency (e.g. means) or other basic estimates (e.g. regression coefficient) AND variation (e.g. standard deviation) or associated estimates of uncertainty (e.g. confidence intervals)
☐	☒	For null hypothesis testing, the test statistic (e.g. F, t, r) with confidence intervals, effect sizes, degrees of freedom and P value noted<br>*Give P values as exact values whenever suitable.*
☒	☐	For Bayesian analysis, information on the choice of priors and Markov chain Monte Carlo settings
☒	☐	For hierarchical and complex designs, identification of the appropriate level for tests and full reporting of outcomes
☒	☐	Estimates of effect sizes (e.g. Cohen's d, Pearson's r), indicating how they were calculated

Our web collection on statistics for biologists contains articles on many of the points above.

Software and code

Policy information about availability of computer code

Data collection	Imagelab software (Bio-rad) was used to acquire q-PCR and Western blots results.
Data analysis	Imagelab software (Bio-rad) was used to analyze q-PCR and Western blots results.<br>ImageJ and Fiji softwares (NIH) were used for immunofluorescence analysis (including YAP n/c ratio), in vitro scratch wound healing assay, in vivo wound closure and space length/area measures.<br>Photoshop software (Adobe; Version CS3) was used for section composition that cross the entire or half cornea.<br>Prism software (GraphPad; Version 6.0) was used to plot volcano graphs and for statistical analysis.<br>KOBAS software was used to test the statistical enrichment of differential expression transcripts in KEGG pathways.<br>Illustrator (Adobe; Version CS5) was used for graphical figure assembly and to draw the schematic graphs.

For manuscripts utilizing custom algorithms or software that are central to the research but not yet described in published literature, software must be made available to editors and reviewers. We strongly encourage code deposition in a community repository (e.g. GitHub). See the Nature Research guidelines for submitting code & software for further information.

Data

Policy information about availability of data

All manuscripts must include a data availability statement. This statement should provide the following information, where applicable:

- Accession codes, unique identifiers, or web links for publicly available datasets
- A list of figures that have associated raw data
- A description of any restrictions on data availability

The RNA-seq data were deposited in NCBI Sequence Read Archive (SRA) under the BioProject accession number PRJNA 669218.

Field-specific reporting

Please select the one below that is the best fit for your research. If you are not sure, read the appropriate sections before making your selection.

☒ Life sciences ☐ Behavioural & social sciences ☐ Ecological, evolutionary & environmental sciences

For a reference copy of the document with all sections, see nature.com/documents/nr-reporting-summary-flat.pdf

Life sciences study design

All studies must disclose on these points even when the disclosure is negative.

Sample size	No statistical methods were used to predetermine sample size. All experiments were repeated at least three times with similar results.The experimental animal numbers, replicate numbers or section numbers were indicated in figure legends.
Data exclusions	Few experimental results of RT-qPCR and Western Blotting were excluded due to serious mistakes during these experiments.
Replication	All experiments were replicated at least three times independently with biological and technical replicates,and showed similar results. Experimental variation was indicated in the figures and legends.
Randomization	For all animal and cell experiments, experimental and control animals and cells were randomly allocated.
Blinding	Most experiments and their analysis were performed by the same investigators, and was not blinding. These experiments, including Ki67 and pH3 staining, q-PCR and in vivo wound closure, were performed and analyzed blindly.

Reporting for specific materials, systems and methods

We require information from authors about some types of materials, experimental systems and methods used in many studies. Here, indicate whether each material, system or method listed is relevant to your study. If you are not sure if a list item applies to your research, read the appropriate section before selecting a response.

Materials & experimental systems

n/a	Involved in the study	
☐	☒	Antibodies
☐	☒	Eukaryotic cell lines
☒	☐	Palaeontology and archaeology
☐	☒	Animals and other organisms
☒	☐	Human research participants
☒	☐	Clinical data
☒	☐	Dual use research of concern

Methods

n/a	Involved in the study	
☒	☐	ChIP-seq
☒	☐	Flow cytometry
☒	☐	MRI-based neuroimaging

Antibodies

Antibodies used	All antibodies used (for Western Blotting, immunofluorescence staining and pull-down) in this study were described in the Methods, for the convenience for the readers.
Validation	All primary antibodies used for immunofluorescence staining were tested by controlling for non-primary conditions with the same procedure of the rest. Moreover, we observed compliance of protein subcellular distribution.<br>All antibodies used for Western Blotting and pull-down, we observed a reasonable band height compliant with the predicted molecular weight.

Eukaryotic cell lines

Policy information about cell lines

Cell line source(s)	Human corneal epithelial cells (hCECs) were obtained from BeNa Cuture Collection (Beijing, China).
Authentication	This is a primary cells and can be cultured for about 15 generations, which express corneal epithelial markers.
Mycoplasma contamination	This cells was not tested for mycoplasma contamination.
Commonly misidentified lines (See ICLAC register)	This study did not involve commonly misidentified cell lines.

Animals and other organisms

Policy information about studies involving animals; ARRIVE guidelines recommended for reporting animal research

Laboratory animals	Adult Long Evans rats (4 months; female and male) were housed in a controlled environment with standard conditions of temperature and humidity with a cycle of an alternating 12h light and dark.<br>New Zealand white rabbits (3-5 months; female and male) were housed in a controlled environment with standard conditions of temperature and humidity with a cycle of an alternating 12h light and dark.
Wild animals	This study did not involve wild animals.
Field-collected samples	This study did not involve samples from the field.
Ethics oversight	All animal protocols were approved by the the Laboratory Animal Welfare and Ethics Committee of the Third Military Medical University (Army Medical University), Chongqing, China.

Note that full information on the approval of the study protocol must also be provided in the manuscript.
